# Stress testing reveals gaps in clinic readiness of image-based diagnostic artificial intelligence models

**DOI:** 10.1038/s41746-020-00380-6

**Published:** 2021-01-21

**Authors:** Albert T. Young, Kristen Fernandez, Jacob Pfau, Rasika Reddy, Nhat Anh Cao, Max Y. von Franque, Arjun Johal, Benjamin V. Wu, Rachel R. Wu, Jennifer Y. Chen, Raj P. Fadadu, Juan A. Vasquez, Andrew Tam, Michael J. Keiser, Maria L. Wei

**Affiliations:** 1 Dermatology Service, San Francisco VA Health Care System, San Francisco, CA USA; 2 Department of Dermatology, University of California, San Francisco, San Francisco, CA USA; 3 Department of Pharmaceutical Chemistry, Department of Bioengineering and Therapeutic Sciences, Institute for Neurodegenerative Diseases, and Bakar Computational Health Sciences Institute, University of California, San Francisco, USA; 4 Helen Diller Family Comprehensive Cancer Center, University of California, San Francisco, San Francisco, CA USA

**Keywords:** Translational research, Diagnosis, Medical imaging, Melanoma, Cancer screening

## Abstract

Artificial intelligence models match or exceed dermatologists in melanoma image classification. Less is known about their robustness against real-world variations, and clinicians may incorrectly assume that a model with an acceptable area under the receiver operating characteristic curve or related performance metric is ready for clinical use. Here, we systematically assessed the performance of dermatologist-level convolutional neural networks (CNNs) on real-world non-curated images by applying computational “stress tests”. Our goal was to create a proxy environment in which to comprehensively test the generalizability of off-the-shelf CNNs developed without training or evaluation protocols specific to individual clinics. We found inconsistent predictions on images captured repeatedly in the same setting or subjected to simple transformations (e.g., rotation). Such transformations resulted in false positive or negative predictions for 6.5–22% of skin lesions across test datasets. Our findings indicate that models meeting conventionally reported metrics need further validation with computational stress tests to assess clinic readiness.

## Introduction

In recent proof-of-principle studies, convolutional neural networks (CNNs) have been shown to perform on par with or better than dermatologists for the classification of skin lesions from images^1–5^, offering great promise for improving patient care through human–computer collaboration^6^. These models are especially relevant today for potential use in telemedicine triage^7^ in the setting of the COVID-19 pandemic. In a qualitative study, patients supported the use of artificial intelligence (AI) for skin cancer screening^8^. However, CNN models mislead clinicians when they give incorrect predictions^6^, with potentially serious consequences. Such incorrect predictions may arise from images that are minimally altered^9^, raising concern for model robustness to images taken in variable conditions. Several other critical concerns for real-world use, such as discrimination and calibration, remain unaddressed^10^, and proof of practice for published models has not been demonstrated.

Discrimination and calibration are metrics that determine the practical usability of prediction models^11^. Discrimination, commonly reported as area under the receiver operating characteristic curve (AUROC), measures whether the CNN correctly yields higher risk predictions for malignant lesions than for benign lesions. Researchers commonly evaluate CNNs by their generalizability, i.e., how well their discrimination performance measured on controlled training data correspond to their performance on new and potentially more diverse hold-out test data. Test data selection is critical: CNN performance is significantly lower on independent test datasets, where test data are from a different institution than training data, compared to dependent test datasets, where training and test data are from the same institution^5^. Public benchmark datasets are needed to compare models and assess generalizability, but the few available are manually curated to include only high-quality images^12,13^. Although CNNs may perform comparably with dermatologists on curated benchmark datasets that exclude low-quality images^14,15^, they may not demonstrate the same discrimination performance when applied to real-world, non-curated datasets.

Calibration quantifies how well a CNN can forecast its accuracy, e.g., whether predictions that the model asserts with 90% confidence are correct 90% of the time. Good calibration, i.e., dependable confidence values, is critical for clinical application, since humans are more likely to rely on CNN judgment when CNN confidence is high^6^. However, CNNs tend to be overconfident and require specialized techniques for calibration^16^. Good calibration is also necessary to enable the process of selective prediction, i.e., soliciting human intervention in place of low-confidence CNN predictions where humans are not initially involved. Only recently have techniques, such as the gambler’s loss approach, been developed to train CNNs with an integrated rejection option to facilitate selective prediction^17^. Few studies have addressed CNN calibration for skin lesion diagnosis^18,19^, and none have addressed selective prediction. This lack of a systematic assessment of how well CNN models are calibrated is a gap in the field. Assessing CNN model calibration across a range of different datasets and image types is one means to address this gap.

In clinical practice, image capture is subject to artifacts such as ink markings and hair as well as variations in zoom, lighting, and focus, yet CNNs have not been rigorously evaluated under these conditions. For example, surgical ink markings can decrease CNN specificity for melanoma diagnosis^20^, and artificially transforming skin lesion images’ zoom, brightness and contrast, and vertical flip have altered the predictions of a CNN with dermatologist-level discrimination^9^. No study, to our knowledge, has systematically assessed this problem. Likewise, no study has examined whether models can give reliable predictions for different images of the same lesion taken in the same setting.

Here, we perform a systematic and rigorous assessment of whether dermatologist-level CNNs, which match or exceed dermatologists’ discrimination in a research environment but are not specifically prepared for real-world deployment, meet three requirements that determine their generalizability for clinical use: (1) discrimination, (2) calibration, and (3) robustness to real-world variations. We first develop CNN models for melanoma vs nevus classification, addressing selective prediction via two different approaches to model development, including the gambler’s loss approach. We then apply several computational stress tests to assess the CNN models’ discrimination and calibration performance across seven test datasets and systematically test their robustness to variations in image capture, image transformations, and disease classes not seen during training. Five of seven of these test datasets deliberately represent settings different from those in which the training data were collected, allowing us to quantify how generalizable the models are on datasets not seen during training. We find that these CNN models, which successfully match dermatologist performance under conventionally reported metrics, perform worse on non-curated datasets, collected in varying settings, compared to curated benchmark datasets that exclude low-quality images. Moreover, these CNN models fail specific and reproducible tests of calibration and practical robustness to image augmentation, revealing heretofore unreported weaknesses. In summary, we demonstrate the implementation of computational stress tests for assessing the clinic readiness of image-based diagnostic AI models and provide a route forward toward addressing gaps in the readiness of current models.

## Results

### Assessing model discrimination

**Fig. 1:**
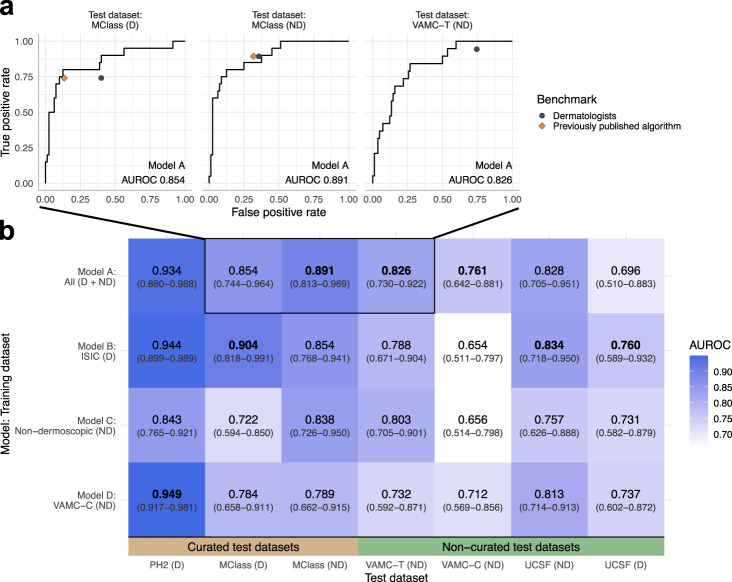
CNN models achieve melanoma discrimination equivalent to or exceeding dermatologists across known and new benchmarks. **a** Model A performs comparably to mean of dermatologists (gray circles) and previously published algorithms^14,15^ (orange diamonds) by ROC curves on the external MClass-D and MClass-ND benchmarks and our VAMC-T benchmark. No previous algorithm has been evaluated on VAMC-T. **b** AUROC is shown for each ensemble model and each benchmark, with darker shades corresponding to higher values. Labels show AUROC values and 95% confidence intervals, with highest per test dataset in bold. ROC curves from (**a**) are boxed. Differences in AUROC between models were not statistically significant. Abbreviations: AUROC area under the receiver operating characteristic curve, CNN convolutional neural network, D Dermoscopic, ISIC International Skin Imaging Collaboration, MClass Melanoma Classification Benchmark, ND Non-dermoscopic, PH2 Hospital Pedro Hispano, ROC Receiver operating characteristic, UCSF University of California, San Francisco, VAMC-C Veterans Affairs Medical Center clinic, VAMC-T Veterans Affairs Medical Center teledermatology.

We developed CNNs for melanoma vs nevus image classification that demonstrated statistically comparable or better discrimination performance compared to dermatologists and previously published CNN models (Fig. 1 and Supplementary Fig. 1). We then assessed if these numerous models that meet commonly accepted published metrics for model performance are robust to systematic and rigorous testing for clinic readiness. Below, we report the results for a representative model, Model A. Results were similar for our other models, and are reported in Supplementary Note 1.

### Assessing model calibration

**Fig. 2:**
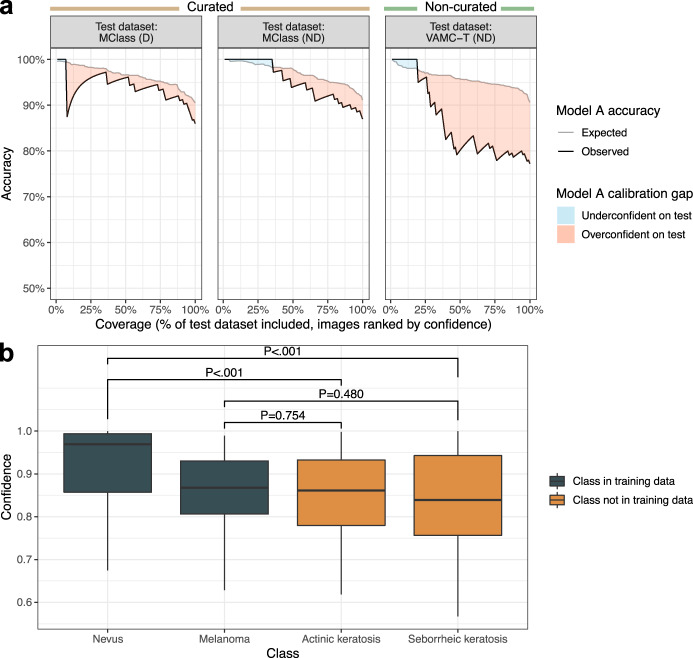
Calibration on development dataset does not generalize to benchmark test datasets. **a** Response rate accuracy curves showing expected accuracy (i.e., accuracy on validation dataset, gray line) and observed accuracy (black line) are plotted against coverage, or the percentage of the test dataset evaluated, with test images ranked by descending Model A prediction confidence. Different values of coverage were obtained by varying the confidence threshold across the range of confidences for test dataset predictions, such that only predictions with confidence greater than the threshold were considered. Accuracy was calculated using a melanoma probability threshold of 0.5, i.e., the predicted class was the class with higher absolute probability. A sharp dip in accuracy from 100% to 87.5% was observed at 8% coverage for MClass-D (*n* = 100) because the prediction ranked 8th out of 100 by confidence was incorrect, resulting in accuracy 7/8 = 87.5%. **b** Model A prediction confidence across test images from disease classes encountered during model training (melanoma, nevus) vs those not encountered during training (actinic keratosis, seborrheic keratosis; confidences plotted on out-of-distribution images are for a prediction of melanoma). All images are from the ISIC archive. *P*-values from the Wilcoxon rank sum test are shown in text. There is no statistically significant difference in confidence on images with a true diagnosis of melanoma vs actinic keratosis (*P* = 0.754) or seborrheic keratosis (*P* = 0.480). Each boxplot displays the median (middle line), the first and third quartiles (lower and upper hinges) and the most extreme values no further than 1.5 * the interquartile range from the hinge (upper and lower whiskers). Abbreviations: D dermoscopic, ISIC International Skin Imaging Collaboration, MClass Melanoma Classification Benchmark, ND non-dermoscopic, VAMC-T Veterans Affairs Medical Center teledermatology.

Current model development commonly omits model calibration. Here, we calibrated models on validation datasets and show that this procedure improved calibration performance on test datasets by moving predicted accuracy closer to observed accuracy (Supplementary Fig. 2). However, even after this calibration procedure, Model A remained overconfident for all test datasets, more so for non-curated vs curated datasets (Supplementary Fig. 2). Model A calibration was worse on the non-curated VAMC-T compared to the curated MClass-D and MClass-ND test datasets, with 77.2% accuracy observed despite 90.7% accuracy expected (difference −13.4%) (Fig. 2a). We found analogous results for all models and test datasets (Supplementary Fig. 3). The calibration performance, measured by root-mean-square error (RMSE) (range 0–1, 0 indicating perfect calibration), universally worsened across all models when performance on any test dataset was compared to performance on the validation dataset, indicating that current calibration procedures on the development dataset do not generalize appropriately to test datasets (Supplementary Fig. 4).

### Out-of-distribution performance

Few published models assess how a model predicts on lesion types that are not included in the training dataset, known as the out-of-distribution problem for CNNs^21^. Here, we evaluated Model A, trained on melanomas and nevi, with regards to prediction on actinic keratoses and seborrheic keratoses, diagnoses not found in the training dataset. The model appropriately assigned lower confidence to images of actinic keratoses and seborrheic keratoses—classes not seen during training, compared to images of nevi—a class seen during training. However, Model A inappropriately assigned similar confidence to images of actinic keratoses and seborrheic keratoses compared to images of melanoma (Fig. 2b). Likewise, models trained using the gambler’s loss failed to reject images of actinic keratoses and seborrheic keratoses at a greater rate than images of melanoma (Supplementary Fig. 5).

### Selective prediction

We evaluated selective prediction by assessing if a model developed with the gambler’s loss approach^17^ could learn when to opt out of difficult scenarios. We found that gambler ensemble models achieved comparable AUROCs (*P* = 0.665) to standard models, but standard and gambler ensemble models had comparable selective prediction performance as measured by AURRA (area under the response rate accuracy curve) (*P* = 0.327). Thus, the gambler’s loss approach did not provide an advantage over standard model training.

### Assessing robustness to differences in image capture

**Fig. 3:**
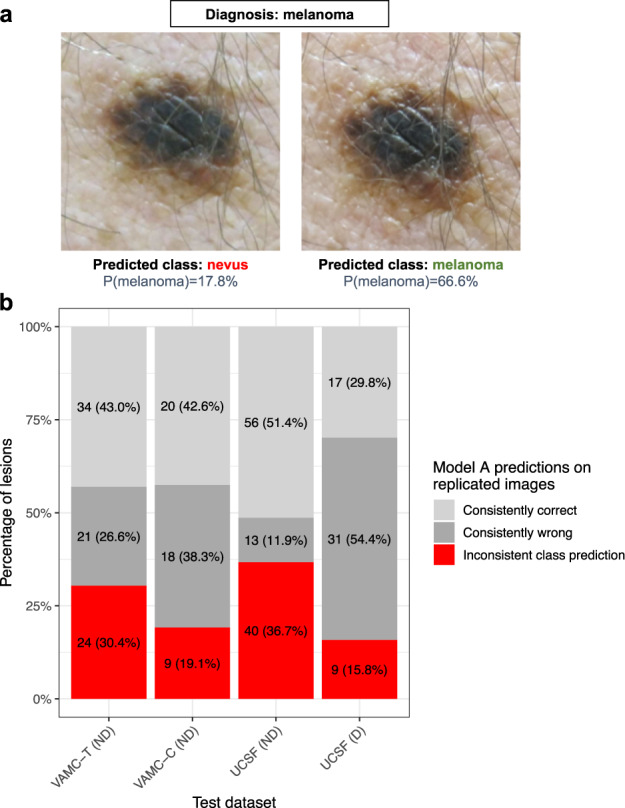
Dermatologist-level CNN models are not robust across different images taken in the same setting. **a** Representative example of Model A predictions on different images of the same lesion taken sequentially during the same clinic session. The predicted probability of melanoma is shown below the predicted class. The decision threshold is model confidence >20.9%, as determined by the operating point at which the model has a sensitivity comparable to dermatologists on VAMC-T, the benchmark containing these images. **b** For the subset of lesions from each test dataset with replicated images, the percentage of lesions is shown grouped by whether predictions across replicated images are all correct, all wrong, or mixed. The decision thresholds are the same as those in (**a**). Only results for non-curated benchmarks are shown, as replicated images were not available for the curated benchmarks. Written consent was obtained for publication of the photographs. Abbreviations: CNN convolutional neural network, D Dermoscopic, ND Non-dermoscopic, UCSF University of California, San Francisco, VAMC-C Veterans Affairs Medical Center clinic, VAMC-T Veterans Affairs Medical Center teledermatology.

In clinics, several views of a skin lesion are typically taken: from different angles, and with different magnifications, such as with and without a dermoscope. This is standard practice for images taken for teledermatology consultation. Thus, we systematically assessed the robustness of our models to such replicated images. Several examples of visually similar replicated images of the same lesion in VAMC-T lead to different Model A predictions (Fig. 3). Twenty-four of 79 (30%) lesions with replicated images in VAMC-T differed in predicted melanoma probability enough to yield inconsistent predictions at the threshold (*t* = 20.9%) matching dermatologists’ management decision sensitivity. Inconsistent predictions for replicated images are present in all test datasets for which replicated images are available (Fig. 3b).

### Assessing robustness to image transformations

**Fig. 4:**
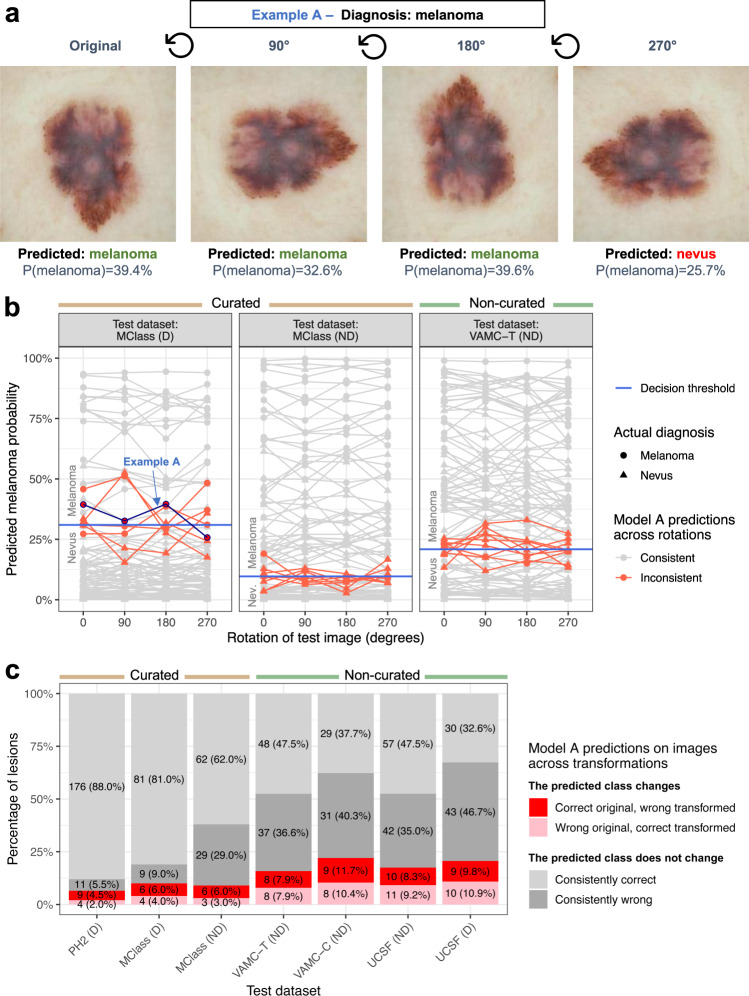
Dermatologist-level CNN models are not robust to image transformations. **a** Representative example of Model A predictions on a melanoma image from MClass-D. The predicted probability of melanoma is shown below the predicted class. The decision threshold is model confidence >31.0%, as determined by the operating point at which the model has a sensitivity comparable to dermatologists for the MClass-D. **b** The predicted melanoma probability across rotations is plotted for each example for each test set. Non-robust images whose predictions cross the decision threshold, selected to match dermatologists’ sensitivity, are plotted in red, robust predictions plotted in gray. The false negative example shown in (**a**) is highlighted by the blue arrow. **c** The percentage of non-robust lesions, assessed over rotations, horizontal flip, brightness, and contrast transformations, is shown as a percentage of the whole dataset for each test dataset. “Consistently correct” and “Consistently wrong” refer to lesions whose prediction is consistent across all transformations. “Correct original, wrong transformed” refers to lesions whose prediction was correct on the original image but was wrong on one or more transformed images. “Wrong original, correct transformed” refers to lesions whose prediction was wrong on the original image but was correct on one or more transformed images. Abbreviations: D Dermoscopic, MClass Melanoma Classification Benchmark, ND Non-dermoscopic, PH2 Hospital Pedro Hispano, UCSF University of California, San Francisco, VAMC-C Veterans Affairs Medical Center clinic, VAMC-T Veterans Affairs Medical Center teledermatology.

Standard model training includes data augmentation with transformations such image rotation and changes in brightness and contrast, but it is unknown whether this data augmentation makes the model robust to these transformations for unseen test images. In a systematic assessment of model performance across image transformations, we found that seemingly banal image changes can result in a false negative prediction for melanoma (Fig. 4a, b). Across the three dermatologist-validated test datasets, Model A’s predicted probability of melanoma varied in response to changes in brightness, contrast, horizontal flip and rotation (median absolute change 0.014, IQR 0.035); this was observed across all models and datasets (Supplementary Fig. 6). Image transformations yielded inconsistent predictions in all test datasets, with 16 of 101 (15.8%) lesions in the VAMC-T test dataset differing in predicted melanoma probability enough to yield inconsistent predictions at the threshold matching dermatologists’ management decision sensitivity (Fig. 4c). Individual lesions often changed predictions in response to multiple independent transformations (Supplementary Fig. 7). Test-time augmentation, i.e., making multiple copies of each test image through data augmentation, having the model make a prediction for each, and then averaging those predictions, did not improve Model A AUROC compared to predictions made on the original test images alone (Supplementary Fig. 8).

## Discussion

Previous reports on CNN models for skin lesion diagnosis established proof of principle for models that can perform comparably to dermatologists in a controlled experimental setting. In contrast, here we focus on their proof of practice, by assessing practical limitations of such models and identifying requirements for clinical deployment. Our results have identified gaps in the development of CNN models despite their meeting dermatologists’ discrimination performance. Crucially, models are not routinely tested for robustness to real-world, non-curated, external datasets, and model development does not encompass selective prediction. To address this gap, we propose and implement actionable computational stress tests to evaluate machine learning tools such as CNNs for image-based diagnosis, to characterize their potential for mistakes and to support safe clinical use.

We propose that models for clinical use should: (1) have adequate discrimination performance on the target population, (2) be well-calibrated and express uncertainty when they are likely to be wrong or unequipped to make a prediction; and (3) generate predictions that are robust to variations in image capture that will be encountered in routine practice. While training models that meet each of these criteria is challenging and outside the scope of this study, we offer suggestions below on how to achieve these goals. Computational stress tests to assess clinic readiness can be applied to CNN models in radiology, ophthalmology, and other fields that rely on medical imaging^22^. A model with physician-level sensitivity and specificity may not necessarily pass these stress tests, as we show that such a model frequently changes its output based on subtle transformations of the input image and erroneously predicts with high confidence on diseases not seen during training. AI tools are already approved for clinical use^23^ without reports of such evaluations, and it is imperative that physicians are aware of these tools’ potential limitations, as faulty AI has been shown to mislead even expert physicians^6^.

We evaluate our models in a clinically meaningful way by comparing them to dermatologists’ management decisions. It is currently difficult to compare CNN models across studies due to proprietary models and, heterogeneous and proprietary test datasets; additional standardized benchmarks are needed to compare model performance^10^.

This study illustrates the problem of selection bias when extrapolating results from high-quality curated datasets to real-world, non-curated datasets. We found higher AUROC on curated vs real-world datasets, though the difference was not statistically significant. For instance, the relatively low AUROC on the UCSF dermoscopic dataset is likely due to the quality of images taken during routine clinical practice, unlike the manually curated and color-adjusted images in ISIC^24^. Interestingly, Model B, trained on dermoscopic images alone, performed comparably to dermatologists for melanoma image classification on non-dermoscopic images (Supplementary Fig. 6), reproducing other groups’ previous findings^14^, which supports the utility of dermoscopic images for optimizing models to classify even non-dermoscopic images. Ultimately, to facilitate generalizability, the population for which the model is meant to be deployed should be represented in the development data.

We show that optimizing calibration performance on the validation dataset was not sufficient for optimization on test datasets, even when the validation data and test data came from the same source (as in Model B/MClass-D and Model D/VAMC-C). Model A is better calibrated for MClass-ND compared to VAMC-T, even though it was calibrated using neither dataset, suggesting that CNNs may better forecast their accuracy on high-quality images in curated benchmark datasets compared to those from non-curated, real-world datasets. To ensure adequate calibration, models will need to be calibrated on samples of the population for which they are to be applied.

The extent to which the models were susceptible to rotation and other transformations was surprising, since these same transformations were part of standard data augmentation during training and the training datasets, likewise, included images of varying quality. Moreover, as in other studies, we had employed pre-training on ImageNet^25^, a large natural image database, which has been shown to improve model robustness and uncertainty estimation^26^. The susceptibility to transformations may occur because there are infinite ways to transform an image (e.g., rotation is continuous), but models can only be exposed to a subset during training no matter how diverse the training dataset. Future work to increase robustness to real-world transformations may involve diversifying training datasets by including multiple images of single lesions captured in different ways, a technique commonly employed when collecting images for teledermatology assessment, as well as specialized computational techniques such as generating adversarial examples during training^27^, modifications to CNN architecture^28^, or leveraging unlabeled examples^29^. It may also help to develop models that can predict based on multiple images of a lesion rather than a single image, though test-time augmentation did not increase AUROC in our study. Along with these strategies to increase robustness, additional standardized metrics of model robustness^30^ are needed to assess readiness for clinical use, to be reported together with discrimination and calibration.

Giving the model the option to abstain from prediction entirely, by training with the gambler’s loss approach^17^, did not improve selective prediction performance compared to standard training, suggesting that currently available machine learning procedures may be inadequate for reliable selective prediction. That Model A predicted melanoma vs benign assignments on actinic keratosis and seborrheic keratosis, lesion classes not seen during training, with similar confidence as on images of melanomas indicated that the out-of-distribution problem remains a potential barrier to clinical use. Future work to allow models to express low confidence or abstain from predicting in cases such as low image quality or disease classes not seen during training may use specialized techniques for this purpose, for example using an “other” class containing various examples not in any training class^21^. Users should be aware that a model that has not been developed specifically to handle the out-of-distribution problem will do its best to blindly predict according to the disease classes it was trained on when it encounters a new disease it was not trained on, with high-confidence predictions potentially leading to false reassurance. An ideal model would first screen images to assess adequacy for decision-making (e.g., based on focus, lighting, presence of artifacts, etc., or similarity to images seen during training) and direct users to retake an image or defer to human experts when appropriate^10^.

Our study population primarily consisted of older, white participants, and we had insufficient data to evaluate generalizability to people with darker skin pigmentation as has been recently done^2^. Additionally, this study did not include all pigmented lesions that may have been suspicious for melanoma or nevus, but rather only those that received a final diagnosis of melanoma or nevus. However, our study aim was not to develop comprehensive models that would readily be deployed in practice, but rather illustrate pitfalls of dermatologist-level CNNs that use a binary classification model. We anticipate these pitfalls would be likely to affect multiclass models (designed to predict more than two diagnoses) as well, given that they are trained using similar CNN architectures, but this was outside the scope of the current study. We tested only one calibration method, temperature scaling, which for CNNs has been shown to be superior to other methods not developed in a deep learning context^16^. Future work could assess additional calibration methods, such as focal loss^31^.

We conclude that CNN models for melanoma image classification that performed comparably to dermatologists nonetheless fail several comparatively straightforward computational stress tests that assess readiness for clinical use. While CNN models are nearly ready to augment clinical diagnostics, the potential for harm can be minimized by evaluating their calibration and robustness to images repeatedly taken of the same lesion and images that have been rotated or otherwise transformed. Our findings support the reporting of model robustness and calibration as a prerequisite for clinical use, in addition to the more common conventions of reporting sensitivity, specificity, and accuracy.

## Methods

### Study approval

This study was approved by the Institutional Review Board of the University of California, San Francisco; written informed consents were obtained, including for the publication of photographs.

### Datasets

We created or acquired multiple non-overlapping skin lesion image datasets for CNN model development (training and validation) and benchmark test datasets (Supplementary Fig. 9 and Supplementary Table 1), only including images of melanoma or nevus. Development datasets were from the San Francisco Veterans Affairs Medical Center (VAMC), the International Skin Imaging Collaboration (ISIC)^32^, DermNetNZ^33^, and the Dermofit Image Library^34^. Test datasets were from the VAMC, the University of California, San Francisco (UCSF), Hospital Pedro Hispano (PH2)^12^, and the dermoscopic and non-dermoscopic Melanoma Classification Benchmarks (MClass-D, and MClass-ND, respectively)^13^. Of the test datasets, we considered MClass-D, MClass-ND, and PH2 to be curated—i.e., containing only images manually selected to be high-quality—and the remainder to be non-curated.

Images from the VAMC composed two datasets: VAMC-T, images of melanoma and nevus lesions in consecutive cases referred for store-and-forward teledermatology and used for testing only (Supplementary Fig. 10); and VAMC-C, images of lesions collected in dermatology clinic, used for both development and testing. Images from UCSF comprised consecutively biopsied lesions from dermatology clinics. Dataset details are in Supplementary Tables 2 and 3. We used a separate dataset from ISIC comprising 132 actinic keratoses and 1518 seborrheic keratoses to evaluate CNN confidence on image classes not seen during training.

The VAMC and UCSF datasets contain lesions for which multiple images were taken during the visit, e.g., from different perspectives, which we denote “replicated images”.

### Model development

We standardized model development by using ImageNet^25^ pre-trained CNNs with the SE-ResNet-50^35,36^ architecture, consistent with previous studies^3,4^. Using five-fold cross-validation we developed four CNN ensemble models (which we shall refer to as Models A–D), each trained using one of four combinations of development data: (A) all images, (B) dermoscopic (magnified) images only, (C) non-dermoscopic images only, and (D) VAMC-C (non-dermoscopic) only. Ensemble model predictions were calculated as the average of the five cross-validation model predictions. We completed two sets of experiments with “standard” models trained using the standard binary cross-entropy loss, and “gambler” models trained with the modified gambler’s loss function^17^, which we hypothesized would improve selective prediction performance. The gambler’s loss function allows a model to opt out of predicting for examples on which it has low confidence. We calibrated each model using its validation dataset with temperature scaling, a method for calibrating neural networks^16^. All predicted probabilities shown are post-calibration. We assessed calibration performance using ℓ_2_ calibration error, or root-mean-square error (RMSE), and difference between expected and observed accuracy on the response rate accuracy (RRA) curve^37^. We assessed selective prediction performance using area under the RRA curve (AURRA)^37^. We detail how to interpret the RRA curve and AURRA in Supplementary Fig. 11. Details on model development are in Supplementary Note 2.

### Dermatologist benchmarks

Previously, the MClass-D and MClass-ND datasets were evaluated by 157 and 145 German academic dermatologists, respectively, with a range of experience levels. They did so via an online questionnaire wherein, “for each image, the participant was asked to make a management decision: (a) biopsy/further treatment or (b) reassure the patient”^13^. Separately, we recruited an independent group of attending US board-certified dermatologists to evaluate the VAMC-T benchmark, likewise using an online questionnaire (REDcap)^38,39^. Fourteen dermatologists completed the VAMC-T survey. Nine (64.3%) reported >10 years of experience, 3 (21.4%) 4–10 years, and 2 (14.3%) <4 years, respectively. Eight (57.1%) reported an academic practice setting. We manually square-cropped each of the 101 skin lesion images to exclude structures outside the lesion while maintaining original resolution. When more than one image of a lesion was available (e.g., different angles), we included 1–2 images that best represented it, based on subjective quality. We removed all patient history and clinical metadata. Participants were informed that all lesions were either nevus or melanoma. For each lesion, the participants were asked for their management and diagnostic decisions: (1) for possible biopsy and (2) nevus vs melanoma.

### Statistical analysis

The main discrimination outcome measures were AUROC for comparing models and Youden index and F1 score for comparing models and dermatologists. Secondary outcomes were area under the precision recall curve (AUPR) for models and ROC area for dermatologists^3^. We used the two-tailed one-sided *t*-test to test the difference in Youden index (sensitivity + specificity − 1; 0–100%) and F1 score (harmonic mean of precision and recall; range, 0–1) between the CNN model and dermatologists. Findings were considered significant at *P* < 0.05.

We computed confidence intervals for AUROC using the DeLong method^40^. To test for differences in AURRA, we used the Wilcoxon signed rank test and for differences in AUROC and confidence, the Wilcoxon rank sum test. We used R, version 4.0.0^41^, for statistical analysis.

### Reporting summary

Further information on research design is available in the Nature Research Reporting Summary linked to this article.

## Supplementary information

Supplementary Information

Reporting Summary

## Data Availability

The ISIC dataset is available at https://www.isic-archive.com/. The PH2 dataset is available at https://www.fc.up.pt/addi/ph2%20database.html. The Melanoma Classification Benchmark is available at https://skinclass.de/mclass/. The DermNetNZ dataset is available for licensing at https://dermnetnz.org/. The Dermofit dataset is available for licensing at https://licensing.edinburgh-innovations.ed.ac.uk/i/software/dermofit-image-library.html. The UCSF and VAMC datasets analyzed during the current study are not publicly available under our Institutional Review Board as they are considered protected health information and cannot be made available to researchers outside this study.

## Code availability

Study code can be made available upon reasonable request.
