# Supplementary material for: Stress testing reveals gaps in clinic readiness of image-based diagnostic artificial intelligence models
Source: NPJ Digit Med. 2021 Jan 21;4:10. doi: 10.1038/s41746-020-00380-6 (PMC7820258; doi:10.1038/s41746-020-00380-6)
Supplement: Supplementary file 1 — Supplementary Information [file 41746_2020_380_MOESM1_ESM.pdf]

Supplementary Information

Supplementary Note 1.
Supplementary Note 2.

Supplementary Table 1. General Characteristics of Included Datasets
Supplementary Table 2. Characteristics of Development Datasets
Supplementary Table 3. Characteristics of Test Datasets
Supplementary Table 4. Discrimination Performance of Model A Versus Dermatologists

Supplementary Figure 1. Standard CNN Models Show Discrimination Performance Comparable to Dermatologists
Supplementary Figure 2. Model Calibration Procedure Decreases Gap Between Predicted and Observed Accuracy
Supplementary Figure 3. CNN Models Are Overconfident on Test Data Despite Model Calibration Procedure on Validation Data
Supplementary Figure 4. Model Calibration Procedure on Validation Data Does Not Achieve Expected Performance on Test Data
Supplementary Figure 5. Gambler CNN Models Fail to Reject Out-of-Distribution Classes
Supplementary Figure 6. CNN Model Predictions Change in Response to Image Transformations
Supplementary Figure 7. Images Become Misclassified Due to Some but Not All Transformations
Supplementary Figure 8. Test-time Augmentation Does Not Improve Discrimination Performance
Supplementary Figure 9. Dataset Split for Model Development and Testing
Supplementary Figure 10. Flow Diagram for Development of VAMC-T Test Dataset
Supplementary Figure 11. Response Rate Accuracy Curves Measure Selective Prediction
Supplementary Figure 12. Gambler CNN Models Show Discrimination Performance Comparable to Dermatologists
Supplementary Figure 13. Some Individual Dermatologists Exceed CNN Model Discrimination Performance for Management Decisions
Supplementary Figure 14. Some Individual Dermatologists Exceed CNN Model Discrimination Performance for Diagnostic Decisions

Supplementary References.

Supplementary Note 1

CNN performance was comparable to dermatologists. To conduct our experiments, we first developed CNNs with dermatologist-level discriminative performance (Supplementary Fig. 9). We tested each standard CNN ensemble model (Models A-D) on seven hold-out test datasets and measured the resulting AUROC (Fig. 1, Supplementary Fig. 1). Model A or B (trained on all data or dermoscopic only, respectively) achieved the highest AUROC for each test dataset except PH2; there was no statistically significant difference between Model A and B AUROCs across test datasets (P>.99). Standard and gambler ensemble models had comparable selective prediction performance as measured by AURRA (P=.327), despite the hypothesis that the gambler would learn to successfully opt out of difficult scenarios. Thus, we report results of Model A (trained on all images) with the simpler standard training procedure in the main figures; gambler ensemble models achieved comparable AUROCs (P=.665) (Supplementary Fig. 12).

At model decision thresholds selected to match dermatologists' management decision sensitivity, in terms of Youden index and F1 score, Model A exceeded dermatologists' performance on the curated MClass-D (CNN sensitivity/specificity 75.0%/90.0% vs dermatologists 74.1%/60.0%; F1 P<.001; Youden P<.001) and on the new non-curated VAMC-T (teledermatology) benchmark (CNN sensitivity/specificity 94.7%/46.3% vs dermatologists 94.3%/25.4%; F1 P<.001; Youden P<.001) (Supplementary Table 4). Model A showed statistically comparable discrimination to dermatologists on the curated MClass-ND by the Youden index (P=.169; CNN sensitivity/specificity 90.0%/62.5% vs dermatologists 89.4%/64.4%) but statistically lower discrimination by the F1 score (P<.001). A number of individual dermatologists surpassed Model A's ROC curve (Supplementary Fig. 13). Model A's sensitivity and specificity were comparable to those of CNN models previously published by an independent group (previous CNN sensitivity/specificity 74.1%/86.5% and 89.4%/68.2% for the MClass dermoscopic and non-dermoscopic datasets, respectively).[1,2] Model A showed statistically comparable AUROCs on non-curated compared to curated test datasets (0.778 [0.063] vs 0.893 [0.040], P=.057). For diagnostic decision-making on VAMC-T, Model A's sensitivity and specificity surpassed dermatologists (Supplementary Fig. 14).

Supplementary Note 2

CNN Architecture

Experiments were conducted using the SE-ResNet-50[3,4] CNN architecture and the PyTorch machine learning library.[5] ImageNet[6] pre-trained model weights were loaded from https://github.com/moskomule/senet.pytorch.

ISIC Training Dataset Details

The International Skin Imaging Collaboration (ISIC)[7] training dataset consisted of the following datasets: 2018 JID Editorial Images, HAM10000,[8] MSK-1, MSK-2, MSK-3, MSK-4, UDA-1, UDA-2. The SONIC dataset was excluded given the presence of colored patches which may confound diagnosis. All images in the dermoscopic Melanoma Classification Benchmark were excluded from the ISIC training dataset to avoid overlap between model development and test datasets.

VAMC and UCSF Test Dataset Details

79.4% of lesions from VAMC and UCSF had "replicated" images photographed from the same lesion in the same encounter. These replicated-image cases had a median of two images per lesion (IQR 2, range 2-14). A.T.Y. manually reviewed each of these lesions while blinded to the diagnosis and selected the subjective best quality image to be included in the corresponding test dataset, such that images in each test dataset represent unique lesions. This exclusion of replicated images was for the purpose of computing model sensitivity and specificity in regard to lesions rather than images. All replicated images were included in the development datasets.

Determination of Model Decision Thresholds for Measuring Robustness

For each of the MClass-D, MClass-ND, and VAMC-T datasets, the decision threshold used to determine correct classification was the minimum probability resulting in a melanoma prediction for which CNN sensitivity on the test dataset matched or exceeded dermatologists' management decision sensitivity on the respective test dataset. For each of the remaining test datasets, the decision threshold was the minimum probability resulting in a melanoma prediction for which CNN sensitivity on the test dataset matched the dermatologists' sensitivity on MClass-D and MClass-ND for dermoscopic and non-dermoscopic test sets, respectively.

Training Dataset Definitions

To balance sampling between datasets of varying sizes during model training, the "all" training dataset, from which Model A was developed, consisted of images from ISIC, Dermofit, DermnetNZ, and VAMC-C, sampled with a 3:1:1:1 ratio during training with the use of sample weights. The "ISIC" training dataset, from which Model B was developed, consisted of images from ISIC only. The "non-dermoscopic" training dataset, from which Model C was developed, consisted of images from Dermofit, DermnetNZ, and VAMC-C, sampled in a 1:1:1 ratio during training. The "VAMC-C" training dataset, from which Model D was developed, consisted of images from VAMC-C only, excluding those present in the test dataset.

Model Training

CNN models were trained on each of four development datasets using five-fold cross-validation with and without the gambler's loss, resulting in 4*5*2=40 total models. In five-fold cross-validation, a development dataset is randomly split into five equally sized datasets, each of which serves in turn as the validation dataset for a model trained on the remaining four datasets, which compose the training dataset. Folds were stratified by class and dataset, such that each fold had approximately the same number of samples per class and dataset. Given the low prevalence of melanomas relative to nevi, for each training dataset, sample weights were computed such that (1) melanomas and nevi were randomly sampled with equal probability during training, and (2) images from different dataset sources would be sampled with the probability indicated by the training dataset definition. The loss function was optimized using stochastic gradient descent with 0.9 momentum. The learning rate was initialized to 0.01 and decayed by 1% after each epoch. Training was stopped if there was no improvement to the best AUROC on the

validation dataset after 40 epochs. CNNs were trained for a median of 58.5 epochs (range 42-119). End-of-training checkpoints were used for model testing.

Data Augmentation

Data augmentation is a standard CNN training procedure that augments the training dataset with selected types of random transformations with the aim of making model predictions more robust to these types of transformations. First, random blur was added to each image during training by randomly resizing the image to NxN pixels, where N was uniformly sampled from [50, 224], and then upsampling the image to 224x224 pixels, the CNN input size. Sequentially, the image was then flipped horizontally with 50% probability and flipped vertically with 50% probability. The brightness, contrast, saturation, and hue were then each changed by a factor uniformly randomly sampled from [0.9, 1.1] separately for each attribute. The image was then randomly rotated between 0 and 360 degrees, randomly translated horizontally and vertically each by a maximum fraction of 0.25, scaled by a factor uniformly randomly sampled from [0.7, 1.3], and randomly sheared horizontally between 0 and 20 degrees. For model testing, we performed data augmentation for the transformation robustness analysis described in the main text and for assessing discrimination performance with test-time augmentation (Supplementary Fig. 8). All test transformations were performed independently on the original image, e.g. images rotated to 180 degrees were rotated directly from the original, not from 90 degrees.

Image Normalization

Data normalization is a standard technique used during CNN training and evaluation to counter mathematical imprecision and improves a CNN's ability to learn from its inputs. We employ the standard scaler approach, which scales data inputs to have zero mean and unit variance. We refer to the mean and standard deviation by which this scaling is accomplished as "normalization parameters." For each training dataset, the per-channel mean and standard deviation over all pixel values were calculated using the entire source dataset. During training, following data augmentation each image was normalized per-channel by subtracting the mean and dividing by the standard deviation calculated from its respective dataset. This dataset-specific normalization was done to minimize dataset-specific bias learned during training. No test images were used in calculating normalization parameters.

During testing, the "ISIC" and "VAMC" models were normalized by their respective normalization parameters used during training. The "all" and "non-dermoscopic" models were normalized by a weighted per-channel mean and standard deviation calculated over the respective training datasets. For example, for the "all" model, the red channel mean was calculated as ½ * the red channel mean of the ISIC training images + ⅙ * the red channel mean of the VAMC training images + ⅙ * the red channel mean of the DermNetNZ training images + ⅓ * the red channel mean of the Dermofit training images. This procedure to compute test-time normalization parameters that best represented the model's training data was done to avoid any information leak that might occur from using the hold-out test datasets to calculate normalization parameters.

Model Calibration

Each model was calibrated using temperature scaling,[9] a standard procedure for calibrating neural networks. Temperature scaling involves optimizing a single parameter, temperature, to maximize calibration performance on the validation set. Different values of temperature increase or decrease uncertainty overall, but temperature does not change the ranking of class predictions and hence does not affect accuracy. We used the implementation in https://github.com/gpleiss/temperature_scaling. For calculating calibration performance via root-mean-square error (RMSE), 15 bins was chosen as it was the published default parameter choice.[9]

Gambler's Loss for Selective Prediction

The *gambler's loss* is a modification to the CNN loss function, without any need for model retraining or changes to model architecture, that has been shown to exceed standard models as well as other methods for selective

prediction.[10] In brief, the gambler's loss involves adding a reject class to the model that represents model abstention from making a prediction. We used the implementation in https://github.com/Z-T-WANG/NIPS2019DeepGamblers.

The hyperparameter *o*, or the payoff for abstention, had previously been described as important to model performance, and thus *o* was tuned during the validation phase.[10] We performed a grid search of *o* between 1.1 and 1.9, as well as initializing *o* to 2 and decaying by a factor of 0.95, 0.98, 0.99, 0.995, or 0.999 per epoch with *o* having a lower bound of 1. Initializing *o* to 2 at the start of training and decaying by a factor of 0.99 at the beginning of each epoch was found to be the most effective regime.

To measure confidence, we first tried defining confidence as 1-P(reject class) or as the highest predicted probability among the non-reject classes (post-softmax layer). Instead, we found that removing the entry corresponding to the reject class in the logits output of the final classification layer, before applying the softmax function, led to better selective prediction performance. In other words, for our experiments, the magnitude of the probability assigned to the reject class was not useful for selective prediction.

Supplementary Tables

Supplementary Table 1. General Characteristics of Included Datasets

Dataset	Location	Description	Number of lesions	Data split
DermNetNZ	New Zealand	Curated non-dermoscopic images collected under varying conditions	1000 melanomas	Train
Dermofit Image Library	United Kingdom	Curated high quality non-dermoscopic images collected under standardized conditions	331 nevi<br>76 melanomas	Train
International Skin Imaging Collaboration (ISIC)	Multiple countries	Curated public archive of standardized dermoscopic images, excluding images in MClass-D	9262 nevi<br>2150 melanomas	Train
San Francisco Veterans Affairs Medical Center clinic (VAMC-C)	United States	Non-curated non-dermoscopic images taken for lesion follow-up or biopsy site documentation in dermatology clinic	561 nevi<br>318 melanomas	Train, Test
San Francisco Veterans Affairs Medical Center teledermatology (VAMC-T)[a]	United States	Non-curated non-dermoscopic images taken by trained imagers for teledermatology, taken from consecutive cases	82 nevi<br>19 melanomas	Test
University of California, San Francisco (UCSF)	United States	Non-curated non-dermoscopic images taken for lesion follow-up or biopsy site documentation in dermatology clinic	104 nevi[b]<br>20 melanomas[c]	Test
Melanoma Classification Benchmark - Non-dermoscopic (MClass-ND)[a]	Nether-lands	Curated public benchmark of high quality non-dermoscopic images, a subset of MED-NODE dataset	80 nevi<br>20 melanomas	Test
Melanoma Classification Benchmark - Dermoscopic (MClass-D)[a]	Multiple countries	Curated public benchmark of high quality dermoscopic images; a random subset of ISIC archive	80 nevi<br>20 melanomas	Test
Hospital Pedro Hispano (PH2)	Portugal	Curated public benchmark dataset of dermoscopic images	160 nevi<br>40 melanomas	Test

[a]Test dataset has been validated by dermatologists
[b]78 have paired dermoscopic image
[c]15 have paired dermoscopic image

Supplementary Table 2. Characteristics of Development Datasets

	**VAMC-C**	**ISIC**	**DermNetNZ**	**Dermofit Image Library**
Image type	Non-dermoscopic	Dermoscopic[a]	Non-dermoscopic	Non-dermoscopic
IMAGES				
Number of images	1776	11,412	1000	407
Melanoma	585 (32.9%)	2150 (18.8%)	1000 (100%)	76 (18.7%)
Nevus	1191 (67.1%)	9262 (81.2%)	0 (0%)	331 (81.3%)
LESIONS				
Number of lesions	879	11,412	1000	407
Melanoma	318 (36.2%)	2150 (18.8%)	1000 (100%)	76 (18.7%)
MIS	*142*	NA	*359*	NA
Invasive	*176*	NA	*641*	NA
Nevus	561 (63.8%)	9262 (81.2%)	0 (0%)	331 (81.3%)
Diagnosis confirmation type	879	11,412	1000	407
Histopathology	879 (100%)	6578 (57.6%)	1000 (100%)	NA
Clinical	0 (0%)	4464 (39.1%)	0 (0%)	NA
Not specified	0 (0%)	370 (3.2%)[b]	0 (0%)	407 (100%)[c]
PARTICIPANTS				
Number of participants	664	NA	NA	NA
Age				
Mean (SD)	67.8 (12.5)	49.1 (16.9)[d]	NA	NA
Min	23	0[d]	NA	NA
25% percentile	62.5	40[d]	NA	NA

	VAMC-C	ISIC	DermNetNZ	Dermofit Image Library
Median	68	50[d]	NA	NA
75% percentile	76	60[d]	NA	NA
Max	93	85[d]	NA	NA
Not stated, count (%)	357 (53.8%)	370 (3.2%)[b,d]	NA	NA
Sex				
Male	561 (84.5%)	NA	NA	NA
Female	22 (3.3%)	NA	NA	NA
Not stated	81 (12.2%)	NA	NA	NA
Race				
White	501 (75.5%)	NA	NA	NA
Black	5 (0.8%)	NA	NA	NA
Asian	16 (2.4%)	NA	NA	NA
Native American	9 (1.4%)	NA	NA	NA
Other	1 (0.2%)	NA	NA	NA
Not stated	132 (19.9%)	NA	NA	NA

Abbreviations: ISIC, International Skin Imaging Collaboration; NA, not available; MIS, melanoma in situ; SD, standard deviation; VAMC-C, Veterans Affairs Medical Center clinic.

[a]Dermoscopic except for 37 non-dermoscopic images of melanoma, which were included as part of the ISIC development dataset

[b]All non-specified values were nevi

[c]Dermofit states that each image has a gold standard diagnosis based on expert opinion (including dermatologists and dermatopathologists), but does offer specific data on how each lesion was diagnosed

[d]Only approximate ages available

Supplementary Table 3. Characteristics of Test Datasets

	**VAMC-C**	**VAMC-T**	**UCSF**	**MClass-D**	**MClass-ND**	**PH2**
Image type(s)	ND	ND	D & ND	D	ND	D
IMAGES						
Diagnosis	283	235	452	100	100	200
Melanoma	81 (28.6%)	56 (23.8%)	84 (18.6%)	20 (20%)	20 (20%)	40 (20%)
Nevus	202 (71.4%)	179 (76.2%)	368 (81.4%)	80 (80%)	80 (80%)	160 (80%)
LESIONS						
Diagnosis	178	101	124	100	100	200
Melanoma	43 (24.2%)	19 (18.8%)	20 (16.1%)	20 (20%)	20 (20%)	40 (20%)
MIS	*29*	*10*	*12*	NA	NA	NA
Invasive	*14*	*9*	*8*	NA	NA	NA
Nevus	135 (75.8%)	82 (81.2%)	104 (83.9%)	80 (80%)	80 (80%)	160 (80%)
Diagnosis confirmation type						
Histopathology	178 (100%)	55 (54.4%)	124 (100%)	47 (47%)	20 (20%)	41 (20.5%)
Melanoma	*43*	*19*	*20*	*20*	*20*	*33*
Nevus	*135*	*36*	*104*	*27*	*0*	*8*
Clinical	0 (0%)	46 (45.5%)	0 (0%)	24 (24%)	80 (80%)	159 (79.5%)
Melanoma	*0*	*0*	*0*	*0*	*0*	*7*
Nevus	*0*	*46*	*0*	*24*	*80*	*152*
Missing	0 (0%)	0 (0%)	0 (0%)	29 (29%)	0 (0%)	0 (0%)

PARTICIPANTS						
	**VAMC-C**	**VAMC-T**	**UCSF**	**MClass-D**	**MClass-ND**	**PH2**
Number of participants	135	89	94	100[b]	NA	NA
Age						
Mean (SD)	64.2 (14.7)	56.5 (15.3)	46.9 (15.1)	42.5 (20.1)[a]	NA	NA
Min	27	23	20	5[a]	NA	NA
25% percentile	58	51	34	30[a]	NA	NA
Median	66	62	46	45[a]	NA	NA
75% percentile	72	66	59	55[a]	NA	NA
Max	93	85	89	85[a]	NA	NA
Missing	0	0	0	19 (19%)	NA	NA
Sex	135	89	94	100[b]	NA	NA
Male	123 (91.1%)	83 (93%)	33 (35%)	44 (44%)	NA	NA
Female	12 (8.9%)	6 (7%)	61 (65%)	37 (37%)	NA	NA
Missing	0	0	0	19 (19%)	NA	NA
Race	135	89	94	100[b]	NA	Not reported, but all from Fitzpatrick skin type II or III
White	109 (80.7%)	56 (63%)	74 (79%)	NA	NA	NA
Black	1 (0.7%)	0 (0%)	0 (0%)	NA	NA	NA
Asian	4 (3.0%)	1 (1%)	6 (6%)	NA	NA	NA

	VAMC-C	VAMC-T	UCSF	MClass-D	MClass-ND	PH2
Native American	0 (0%)	2 (2%)	0 (0%)	NA	NA	NA
Other	0 (0%)	1 (1%)	10 (11%)	NA	NA	NA
Missing	26 (19.3%)	29 (33%)	4 (4%)	NA	NA	NA

Abbreviations: D, dermoscopic; MClass, Melanoma Classification Benchmark; MIS, melanoma in situ; NA, not available; ND, non-dermoscopic; SD, standard deviation; UCSF, University of California, San Francisco; VAMC-C, Veterans Affairs Medical Center clinic; VAMC-T, Veterans Affairs Medical Center teledermatology.

[a]Only approximate ages available

Supplementary Table 4. Discrimination Performance of Model A Versus Dermatologists

	MClass-D		MClass-ND		VAMC-T	
	Model A	Derm	Model A	Derm	Model A	Derm
Youden Index Score, Mean (SD)[a]	0.650	0.341 (0.099)	0.525	0.537 (0.109)	0.411	0.198 (0.150)
F1 Score, Mean (SD)[a]	0.698	0.446 (0.050)	0.529	0.550 (0.070)	0.444	0.374 (0.051)
Sensitivity, % (SD)[a]	75.0	74.1 (9.9)	90.0	89.4 (9.4)	94.7	94.4 (6.7)
Specificity, % (SD)[a]	90.0	60.0 (13.3)	62.5	64.4 (14.3)	46.3	25.4 (20.2)
AUPR	0.688	Not applicable	0.692	Not applicable	0.542	Not applicable
ROC Area, Mean (SD)	Not applicable	Not available	Not applicable	Not available	Not applicable	0.694 (0.075)
AUROC (95% CI)	0.854 (0.744-0.964)	Not applicable	0.891 (0.813-0.969)	Not applicable	0.826 (0.730-0.922)	Not applicable

Abbreviations: AUROC, area under the receiver operating characteristic curve; AUPR, area under the precision recall curve; CI, confidence interval; D, dermoscopic; Derm, mean dermatologists; MClass, Melanoma Classification Benchmark; NA, not applicable; ND, non-dermoscopic; ROC, receiver operating characteristic curve; SD, standard deviation.

[a]Model A standard deviation not applicable

Supplementary Figures

Supplementary Figure 1. Standard CNN Models Show Comparable Discrimination Performance to Dermatologists

ROC curves are shown for each standard CNN model for each test dataset, including the ensemble model (black line) and cross-validation models (colored lines). True positive rate and false positive rate are shown for mean dermatologists (gray circles) and previous algorithms[1,2] (orange diamonds) for datasets where data is available. The circles representing comparisons with dermatologists and previous algorithms are faded apart from the models that offer the fairest comparison, e.g. the MClass test datasets were previously validated using models trained on dermoscopic images from ISIC. AUROC is shown in text for the ensemble model. Abbreviations: AUROC, area under the receiver operating characteristic curve; CNN, convolutional neural network; CV, cross-validation; D, dermoscopic; ISIC, International Skin Imaging Collaboration; MClass, Melanoma Classification Benchmark; ND, non-dermoscopic; PH2, Hospital Pedro Hispano; VAMC-C, Veterans Affairs Medical Center clinic; VAMC-T, Veterans Affairs Medical Center teledermatology.

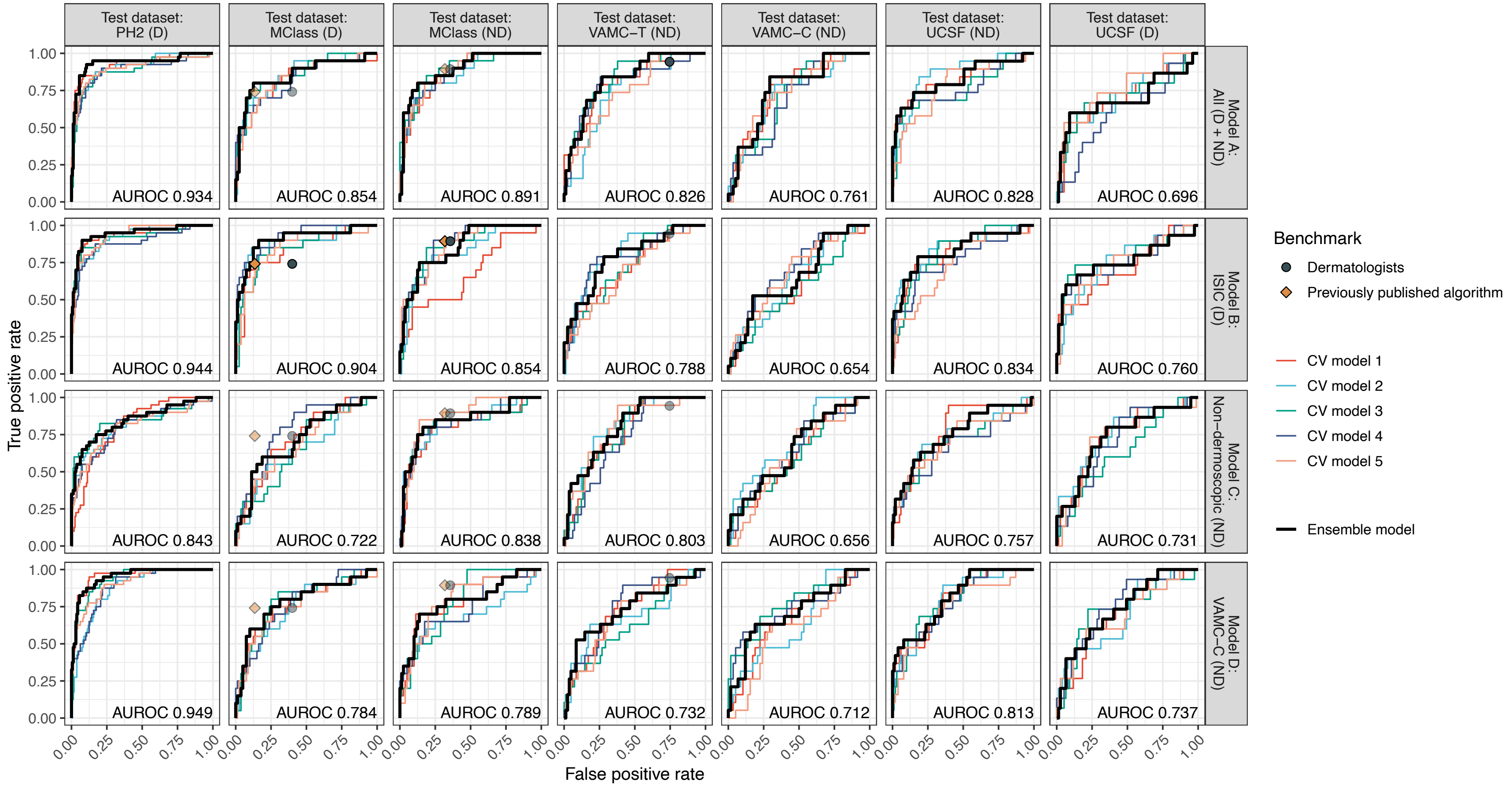

Supplementary Figure 2. Model Calibration Procedure Decreases Gap Between Predicted Accuracy and Observed Accuracy

For the validation dataset and each test dataset, the distribution of prediction confidence, i.e. predicted accuracy, is plotted pre- (*top*) and post-calibration (*bottom*). Each histogram has 15 bins, and the y-axis is the density of points in a bin, scaled to integrate to 1 over all bins (i.e. bin width x density, summed across all bins, is equal to 1). Average accuracy and average confidence over predictions are plotted in the solid and dashed lines, respectively, and should overlap in a perfectly calibrated model. The post-calibration average accuracy and average confidence are not equal for the validation dataset because the objective of the temperature scaling calibration procedure is to minimize RMSE between accuracy and confidence within each of the 15 bins, rather than minimizing RMSE between accuracy and confidence of the entire validation dataset together. Abbreviations: D, dermoscopic; MClass, Melanoma Classification Benchmark; ND, non-dermoscopic; PH2, Hospital Pedro Hispano; RMSE, root-mean-square error; VAMC-C, Veterans Affairs Medical Center clinic; VAMC-T, Veterans Affairs Medical Center teledermatology.

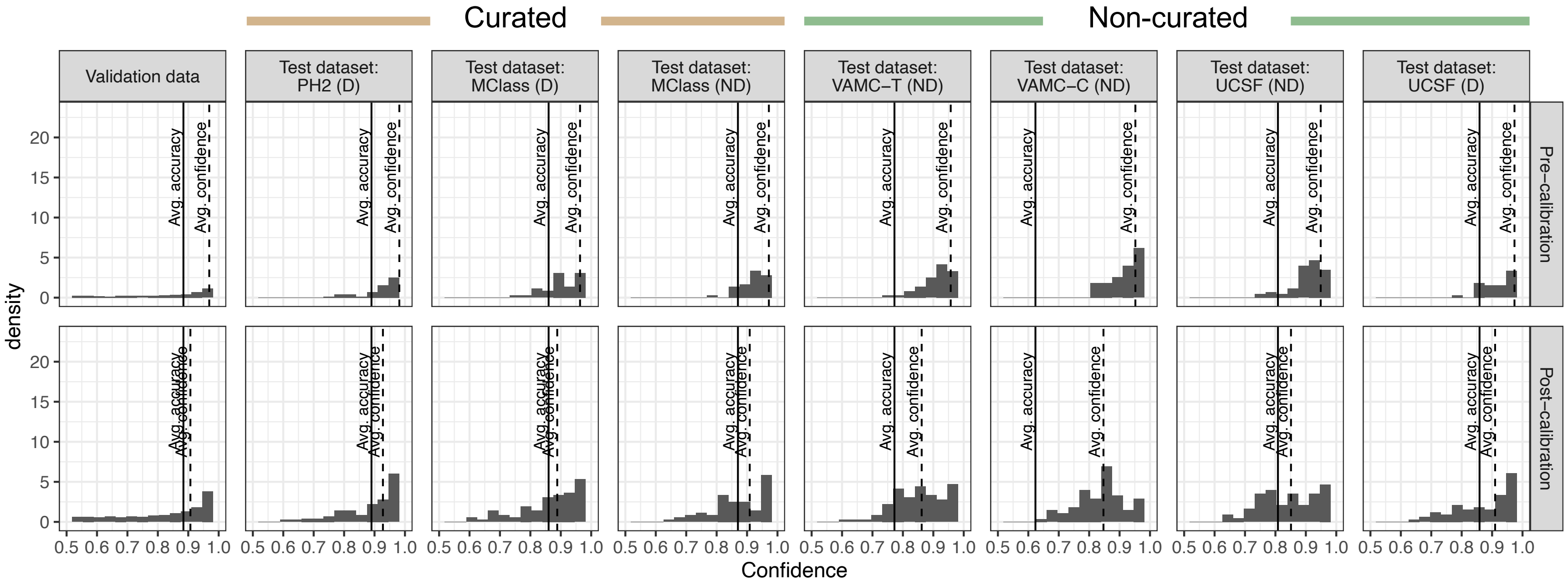

Supplementary Figure 3. CNN Models Are Overconfident on Test Data Even After Model Calibration Procedure on Validation Data

Response rate accuracy curves showing expected accuracy (i.e. accuracy on validation dataset after calibration, gray line) and observed accuracy (black line), i.e. accuracy on test dataset, are plotted against coverage, or the percentage of the test dataset included, with test samples ranked by descending prediction confidence. Different values of coverage were obtained by varying the confidence threshold across the range of confidences for test dataset predictions, such that only predictions with confidence greater than the threshold were included. Accuracy was calculated using a melanoma probability threshold of 0.5, i.e. the predicted class was the class with higher absolute probability. AURRA (range 0-1), the area under the black line, is shown in text for test data. Higher AURRA values indicate higher selective prediction performance. Abbreviations: AURRA, area under the response rate accuracy curve; D, dermoscopic; MClass, Melanoma Classification Benchmark; ND, non-dermoscopic; PH2, Hospital Pedro Hispano; VAMC-C, Veterans Affairs Medical Center clinic; VAMC-T, Veterans Affairs Medical Center teledermatology.

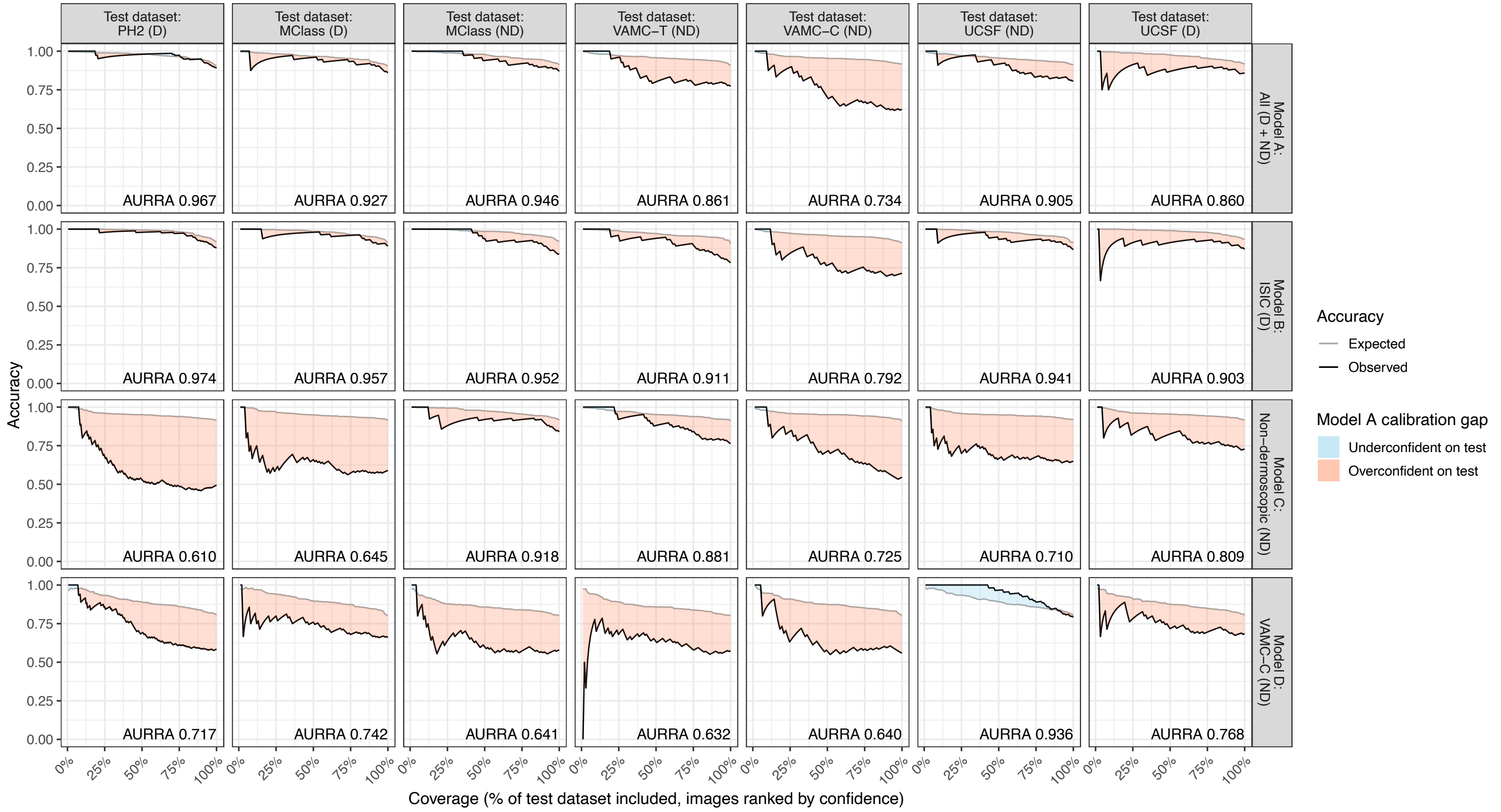

Supplementary Figure 4. Model Calibration Procedure on Validation Data Does Not Achieve Expected Performance on Test Data

Pre- and post-calibration RMSE (range 0-1) is shown for each test dataset and model. Lower values indicate better calibration. The dashed and dotted lines show the validation RMSE (averaged over different folds) and perfect calibration, respectively. Abbreviations: D, dermoscopic; MClass, Melanoma Classification Benchmark; ND, non-dermoscopic; PH2, Hospital Pedro Hispano; RMSE, root-mean-square error; VAMC-C, Veterans Affairs Medical Center clinic; VAMC-T, Veterans Affairs Medical Center teledermatology.

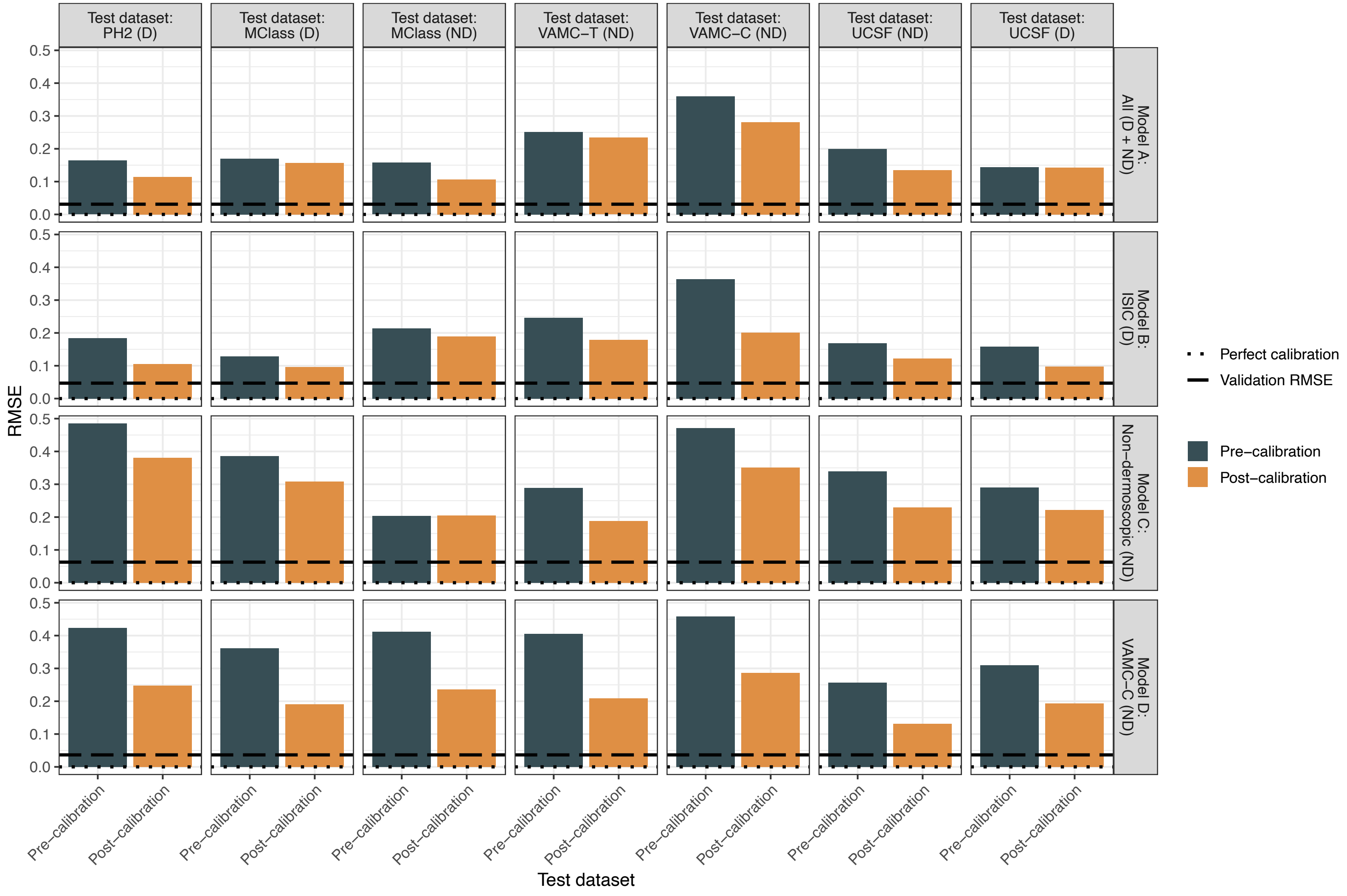

Supplementary Figure 5. Gambler CNN Models Fail to Reject Out-of-Distribution Classes

Plotted for each of the four ensemble gambler models is the probability assigned to the reject class across test images from disease classes encountered during model training (melanoma, nevus) versus those not encountered during training (actinic keratosis, seborrheic keratosis). For each prediction, the probability of melanoma, nevus, and the reject class sums to 1. All test images are from the ISIC archive. Each boxplot displays the median (middle line), the first and third quartiles (lower and upper hinges) and the most extreme values no further than 1.5 * the interquartile range from the hinge (upper and lower whiskers). Abbreviations: D, dermoscopic; ISIC, International Skin Imaging Collaboration; ND, non-dermoscopic; VAMC-C, Veterans Affairs Medical Center clinic.

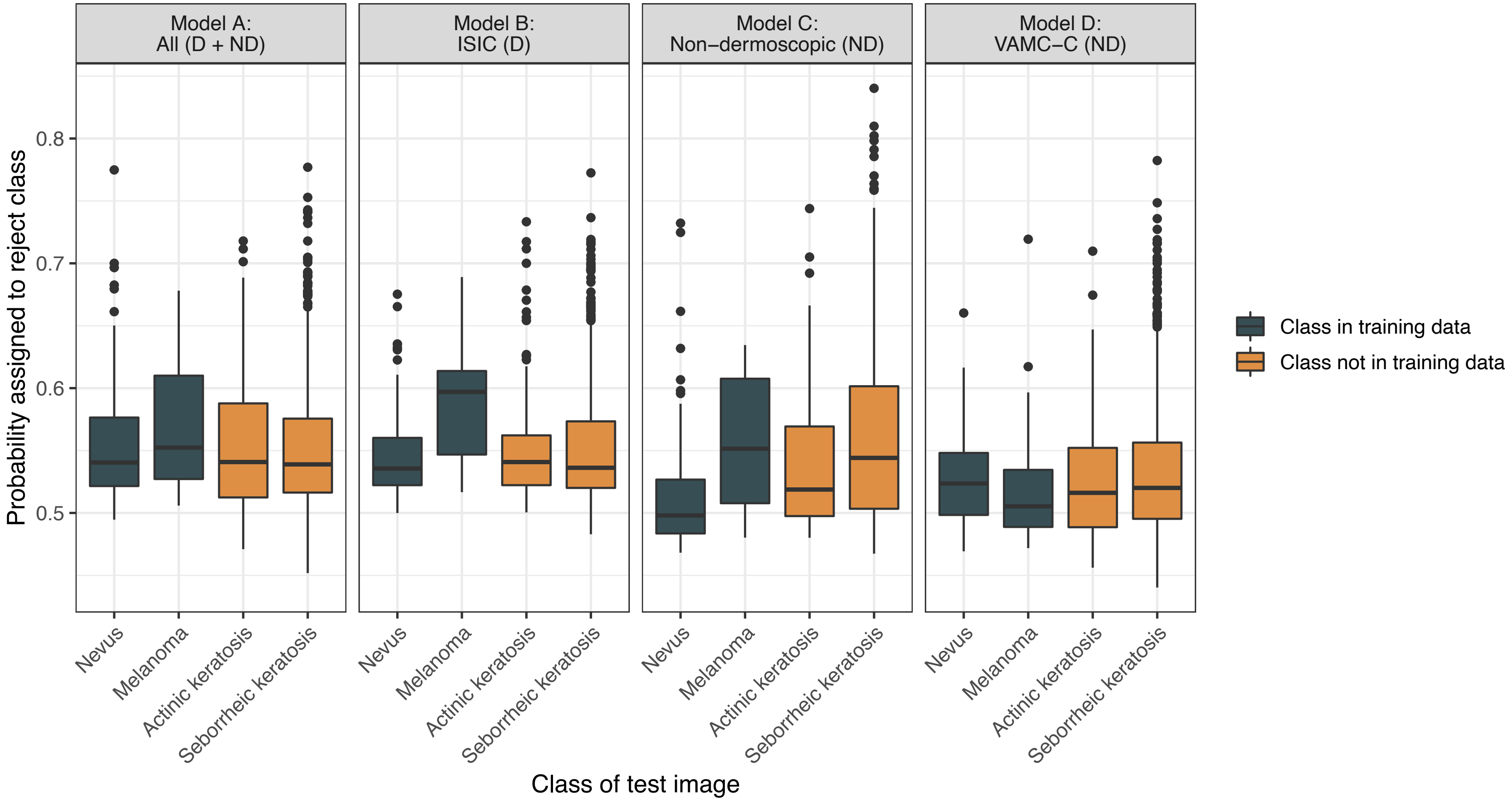

Supplementary Figure 6. CNN Model Predictions Change in Response to Image Transformations

For each standard model and test dataset, boxplots display the distribution across lesions of absolute change in predicted melanoma probability, P(melanoma), with respect to the original image as a result of a given artificial transformation. In the x-axis labels, 0.9 and 1.1 correspond to decreasing and increasing the brightness or contrast by a factor of 0.9 or 1.1, respectively, and 90, 180, and 270 correspond to the degree of rotation. Each boxplot displays the median (middle line), the first and third quartiles (lower and upper hinges) and the most extreme values no further than 1.5 * the interquartile range from the hinge (upper and lower whiskers). Abbreviations: D, dermoscopic; MClass, Melanoma Classification Benchmark; ND, non-dermoscopic; PH2, Hospital Pedro Hispano; VAMC-C, Veterans Affairs Medical Center clinic; VAMC-T, Veterans Affairs Medical Center teledermatology.

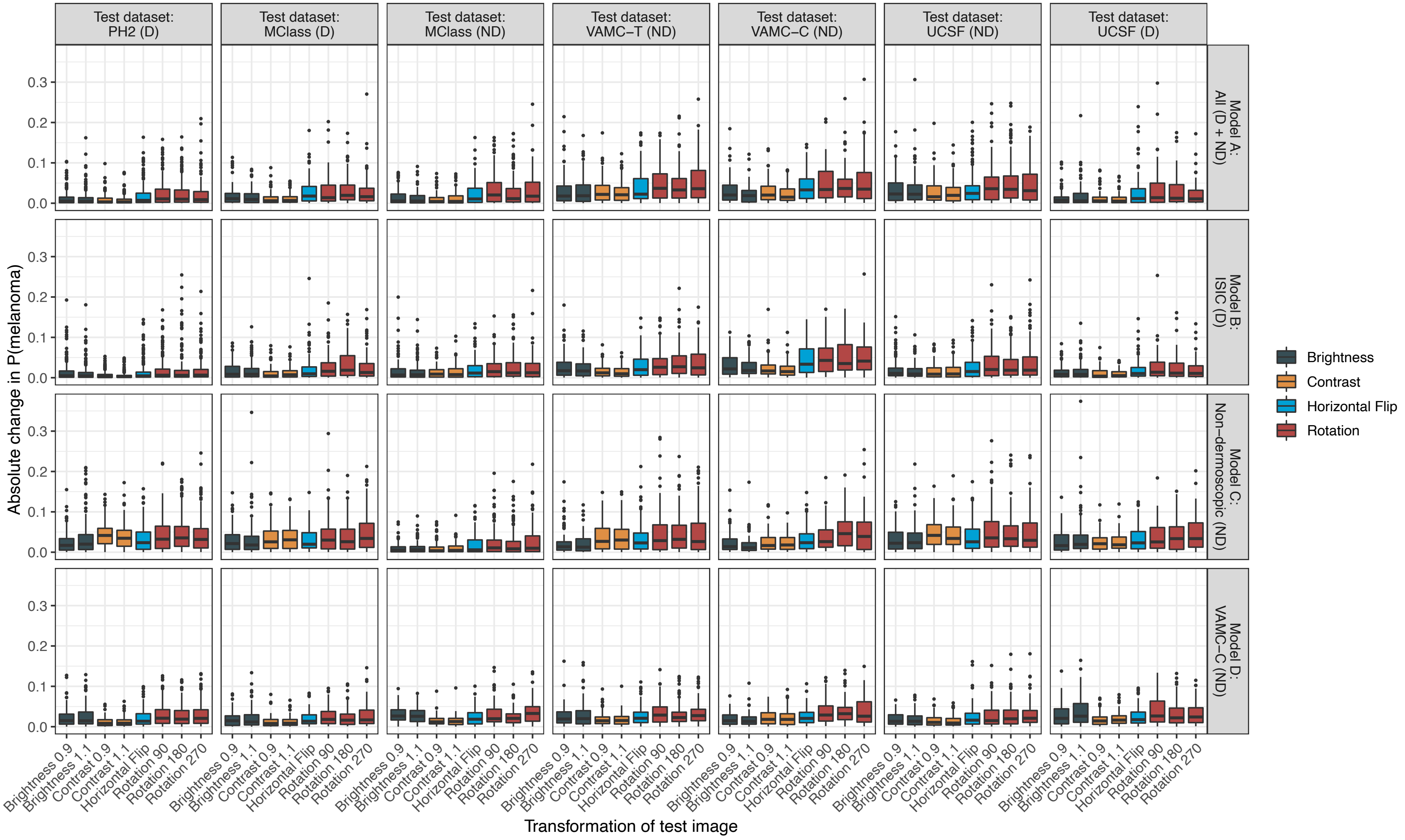

Supplementary Figure 7. Images Become Misclassified Due to Some but Not All Transformations

For each test dataset, examples misclassified due to one or more transformations are shown, with red indicating the transformation(s) leading to misclassification and gray indicating correct classification. For MClass-D, MClass-ND, and VAMC-T, the probability threshold used to determine correct classification was the minimum probability resulting in a melanoma prediction for which CNN sensitivity on the test dataset matched or exceeded dermatologists' decision-to-biopsy sensitivity on the respective test dataset. For each of the remaining test datasets, the probability threshold used to determine correct classification was the minimum probability resulting in a melanoma prediction for which CNN sensitivity on the test dataset matched or exceeded dermatologists' sensitivity for decision-to-biopsy (94.7%) on VAMC-T. Abbreviations: D, dermoscopic; MClass, Melanoma Classification Benchmark; ND, non-dermoscopic; PH2, Hospital Pedro Hispano; VAMC-C, Veterans Affairs Medical Center clinic; VAMC-T, Veterans Affairs Medical Center teledermatology.

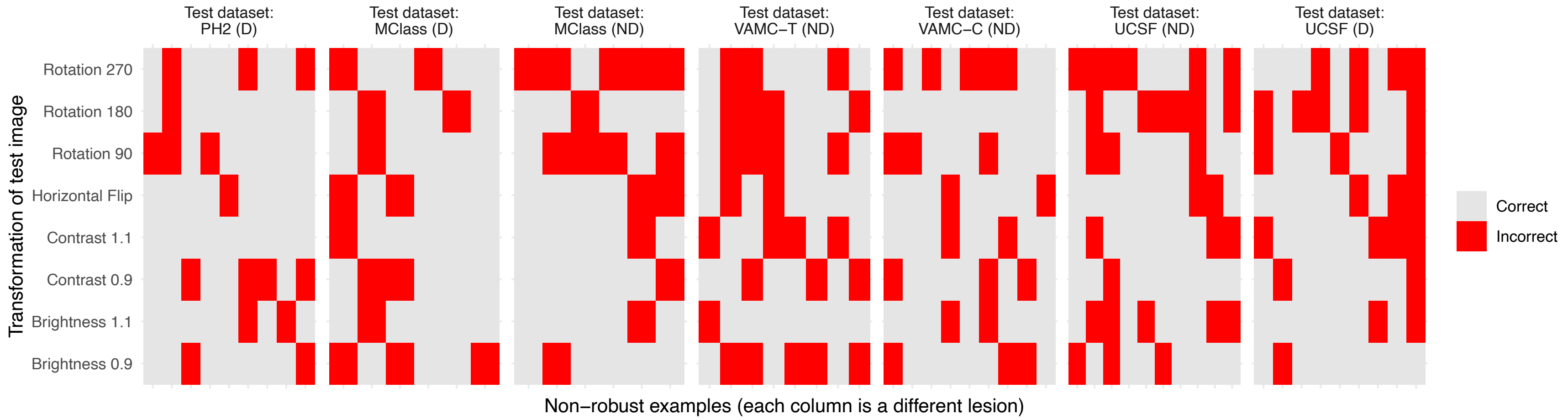

Supplementary Figure 8. Test-time Augmentation Does Not Improve Discrimination Performance

We created 100 augmented copies of each test image using the same random transformations used during training. We then generated ensemble predictions for Model A by averaging its predictions on each of the 100 copies. We then compared Model A's discrimination performance, measured by AUROC, based on these ensemble predictions to its predictions based on the original test images. Abbreviations: AUROC, area under the receiver operating characteristic curve; D, dermoscopic; MClass, Melanoma Classification Benchmark; ND, non-dermoscopic; PH2, Hospital Pedro Hispano; VAMC-C, Veterans Affairs Medical Center clinic; VAMC-T, Veterans Affairs Medical Center teledermatology.

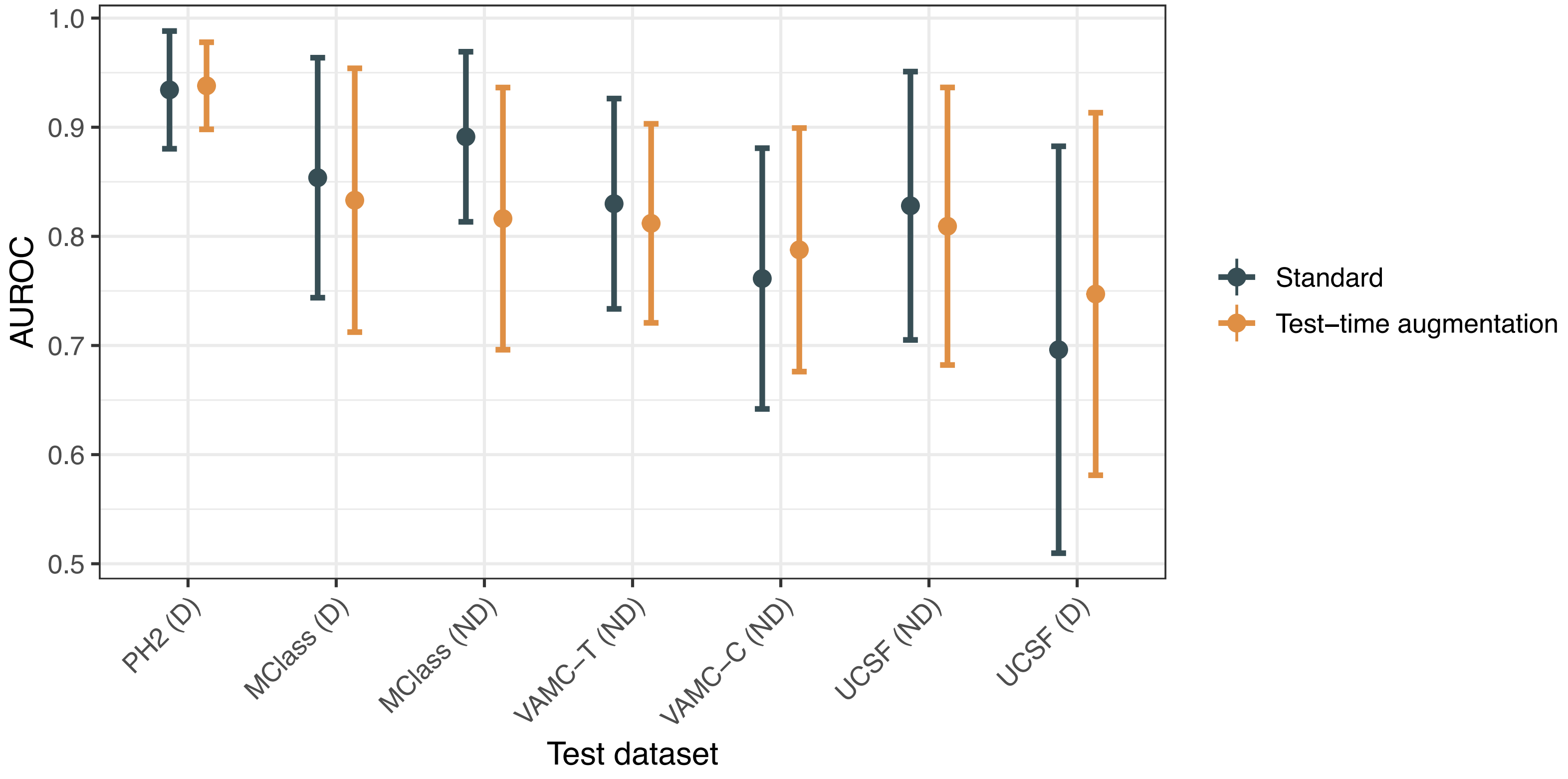

Supplementary Figure 9. Dataset Split for Model Development and Testing

We divided study data into a portion used for model development and a hold-out test (benchmark) dataset used for model assessment. Development data comprised four source datasets, from which we used different combinations to develop ensemble Models A-D, respectively. For clarity, the figure shows development of Model A from development dataset A. Models B-D were analogously developed from development datasets B-D. We randomly split each development dataset into five equally sized folds. We then trained five separate cross-validation models with a different fold (blue) held out in turn and used to calibrate the model; we combined the remaining folds (gray) to train the model. We ensembled the model across its five cross-validation models (across rows) by taking their average for each prediction. Abbreviations: D, dermoscopic; ISIC, International Skin Imaging Collaboration; ND, non-dermoscopic; UCSF, University of California, San Francisco; VAMC-C, Veterans Affairs Medical Center clinic; VAMC-T, Veterans Affairs Medical Center teledermatology. [a]dermatologist-validated; [b]non-overlapping development and test datasets.

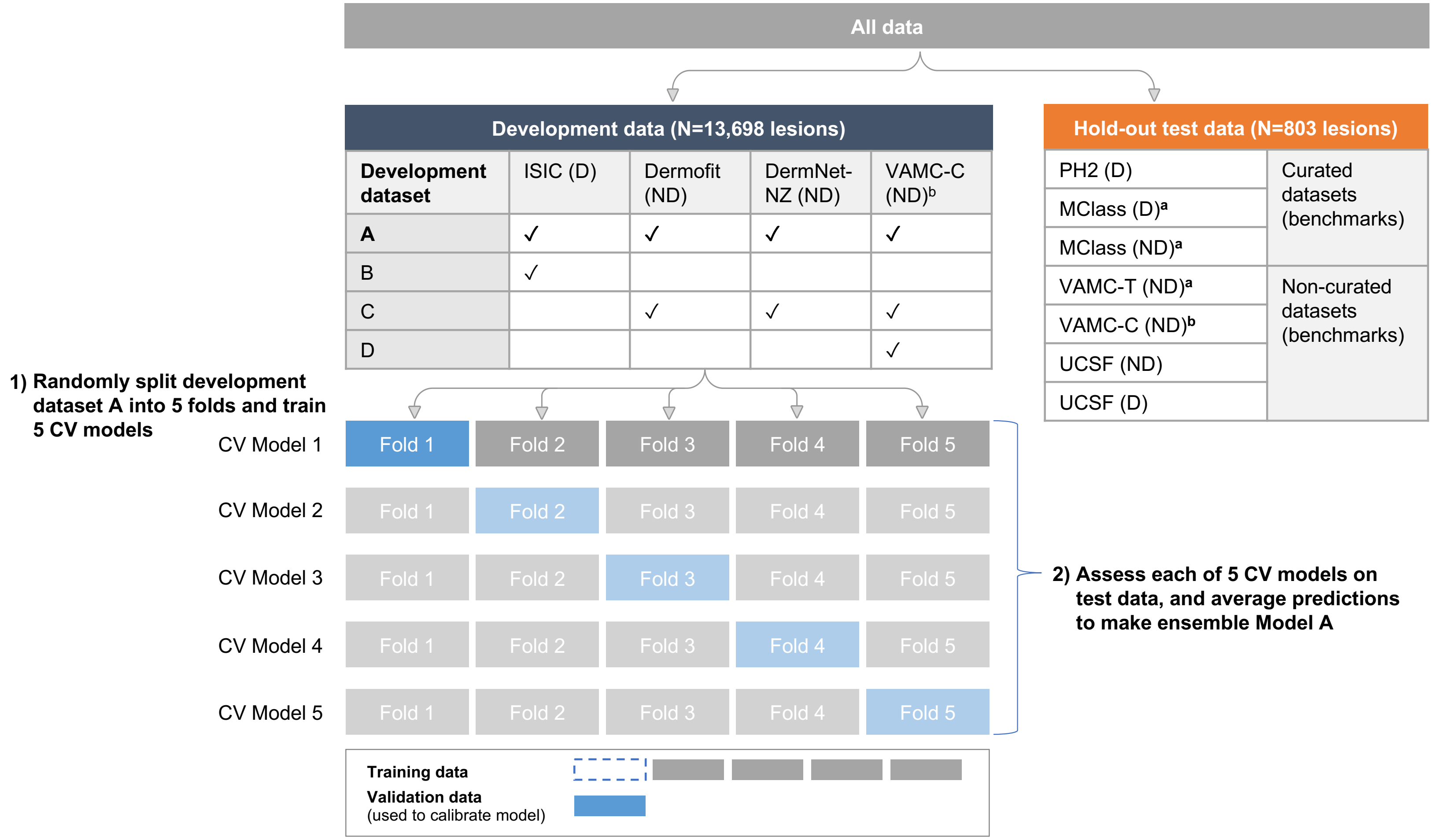

Supplementary Figure 10. Flow Diagram for Development of VAMC-T Test Dataset

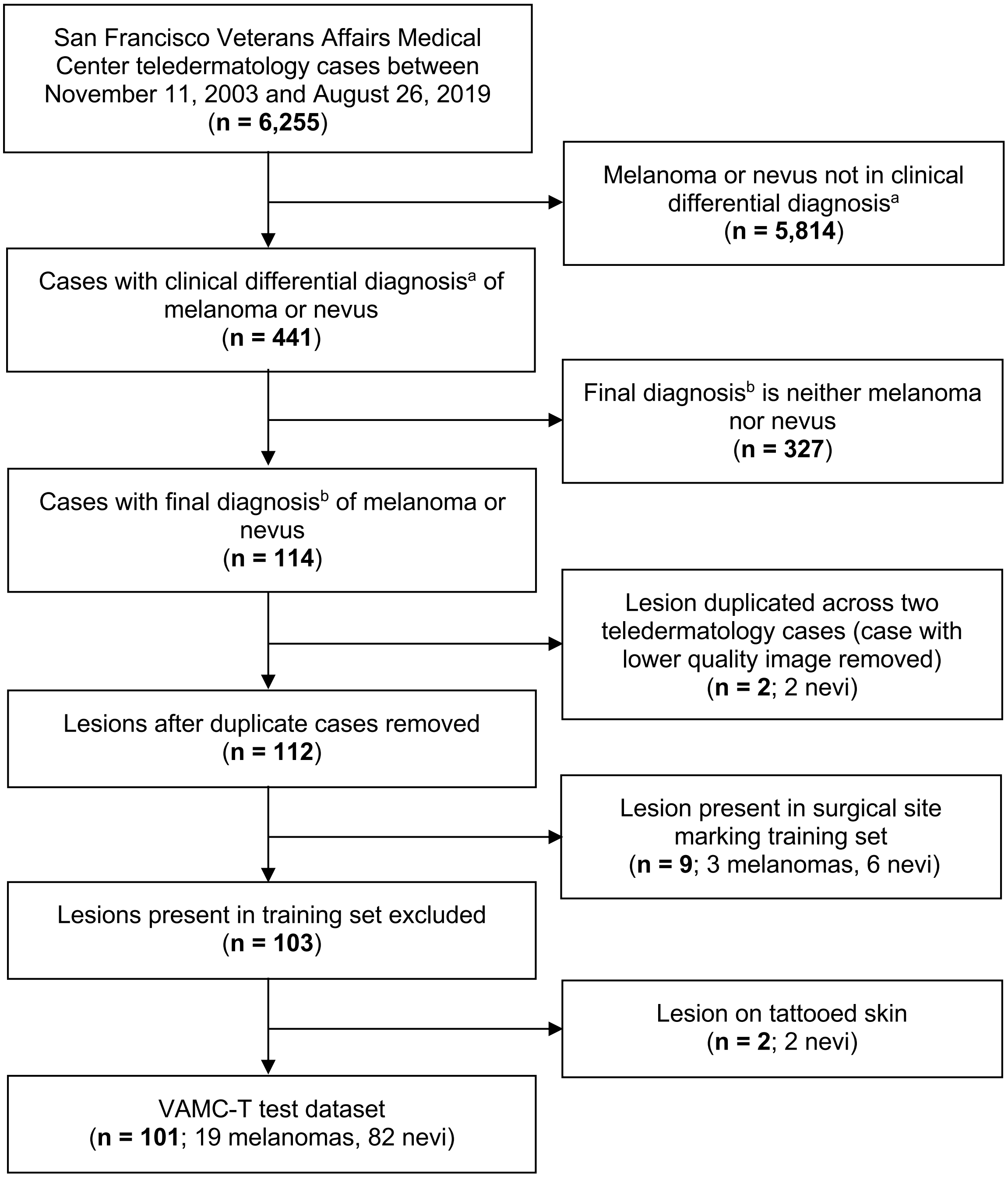

[a]Clinical diagnosis made by reading teledermatologist

[b]Final diagnosis made using histopathology if biopsy done, else clinical diagnosis

Supplementary Figure 11. Response Rate Accuracy Curves Measure Selective Prediction

Two hypothetical models that each achieve 60% accuracy on a test dataset of five examples differ in selective prediction performance, as measured by response-rate accuracy (RRA) curves and area under the RRA curve (AURRA). The RRA curve plots accuracy vs coverage, or the percentage of the test dataset, with examples ranked by confidence, used to calculate the accuracy. The first model (A) appropriately assigns higher confidence to examples it predicted correctly than examples it predicted incorrectly, allowing the model to achieve 100% accuracy if it abstains from predicting on the least confident 40% of samples. This model achieves an AURRA of (1/1 + 2/2 + 3/3 + 3/4 + 3/5)/5 = 0.870, the highest possible AURRA given accuracy on the overall test dataset. The second model (B) inappropriately assigns higher confidence to an incorrectly predicted example than correctly predicted examples, resulting in a lower AURRA of (1/1 + 1/2 + 2/3 + 3/4 + 3/5)/5 = 0.703.

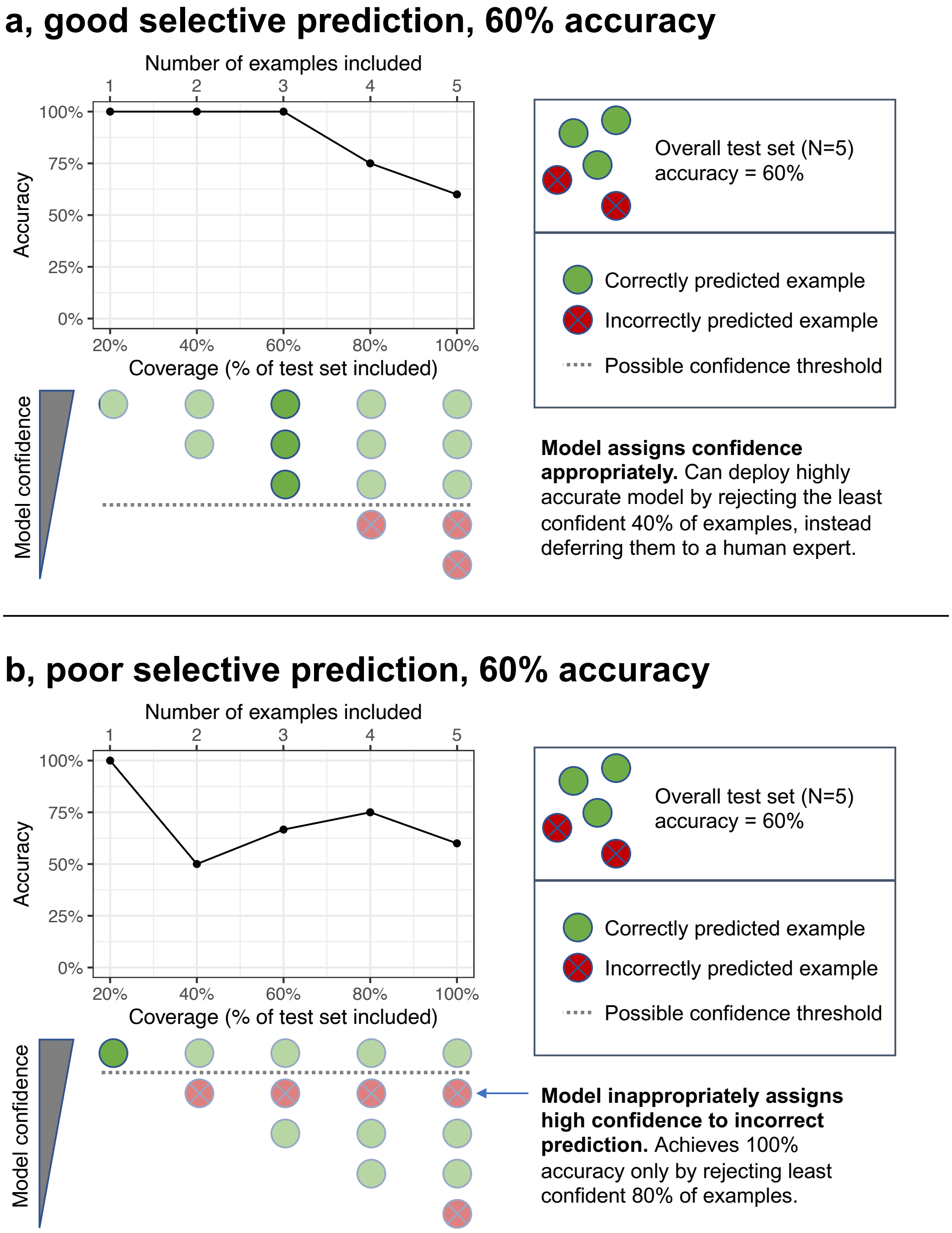

Supplementary Figure 12. Gambler CNN Models Show Discrimination Performance Comparable to Standard CNN Models

ROC curves are shown for each gambler CNN model for each test dataset, including the ensemble model (black line) and cross-validation models (colored lines). True positive rate and false positive rate are shown for mean dermatologists (gray circles) and previous algorithms[1,2] (orange diamonds) for datasets where data is available. The circles representing comparisons with dermatologists and previous algorithms are faded apart from the models that offer the fairest comparison, e.g. the MClass test datasets were previously validated using models trained on dermoscopic images from ISIC. AUROC is shown in text for the ensemble model. Abbreviations: AUROC, area under the receiver operating characteristic curve; CNN, convolutional neural network; CV, cross-validation; D, dermoscopic; ISIC, International Skin Imaging Collaboration; MClass, Melanoma Classification Benchmark; ND, non-dermoscopic; PH2, Hospital Pedro Hispano; VAMC-C, Veterans Affairs Medical Center clinic; VAMC-T, Veterans Affairs Medical Center teledermatology.

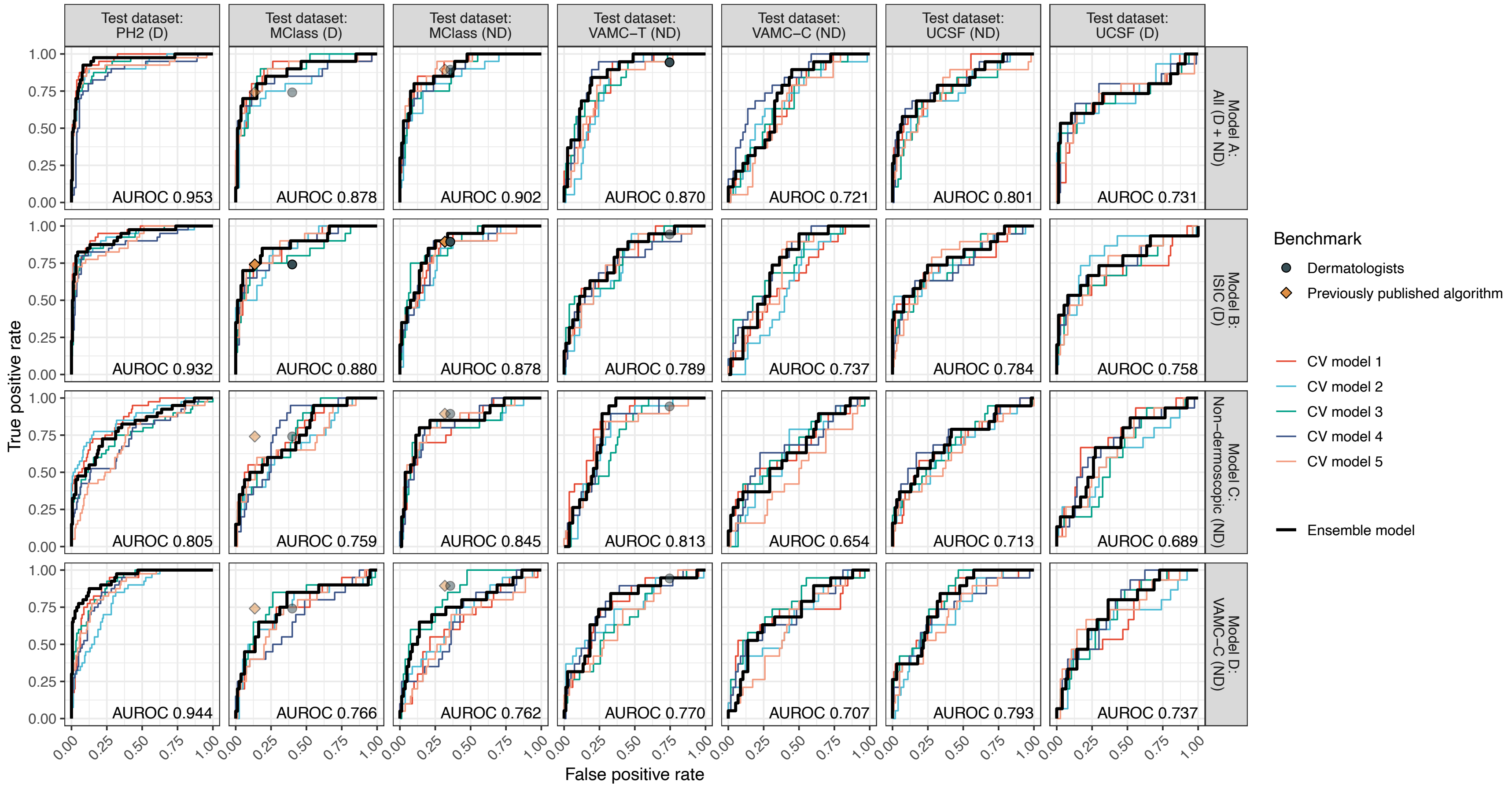

Supplementary Figure 13. Some Individual Dermatologists Exceed CNN Model Discrimination Performance for Management Decision

ROC curves are shown for each standard CNN ensemble model for each dermatologist-validated test dataset. True and false positive rates for management decision (biopsy vs clinical follow-up) are shown for mean of dermatologists (blue circles) and individual dermatologists (gray circles). AUROC is shown in text for the ensemble model. Abbreviations: AUROC, area under the receiver operating characteristic curve; CNN, convolutional neural network; D, dermoscopic; MClass, Melanoma Classification Benchmark; ND, non-dermoscopic; VAMC-T, Veterans Affairs Medical Center teledermatology.

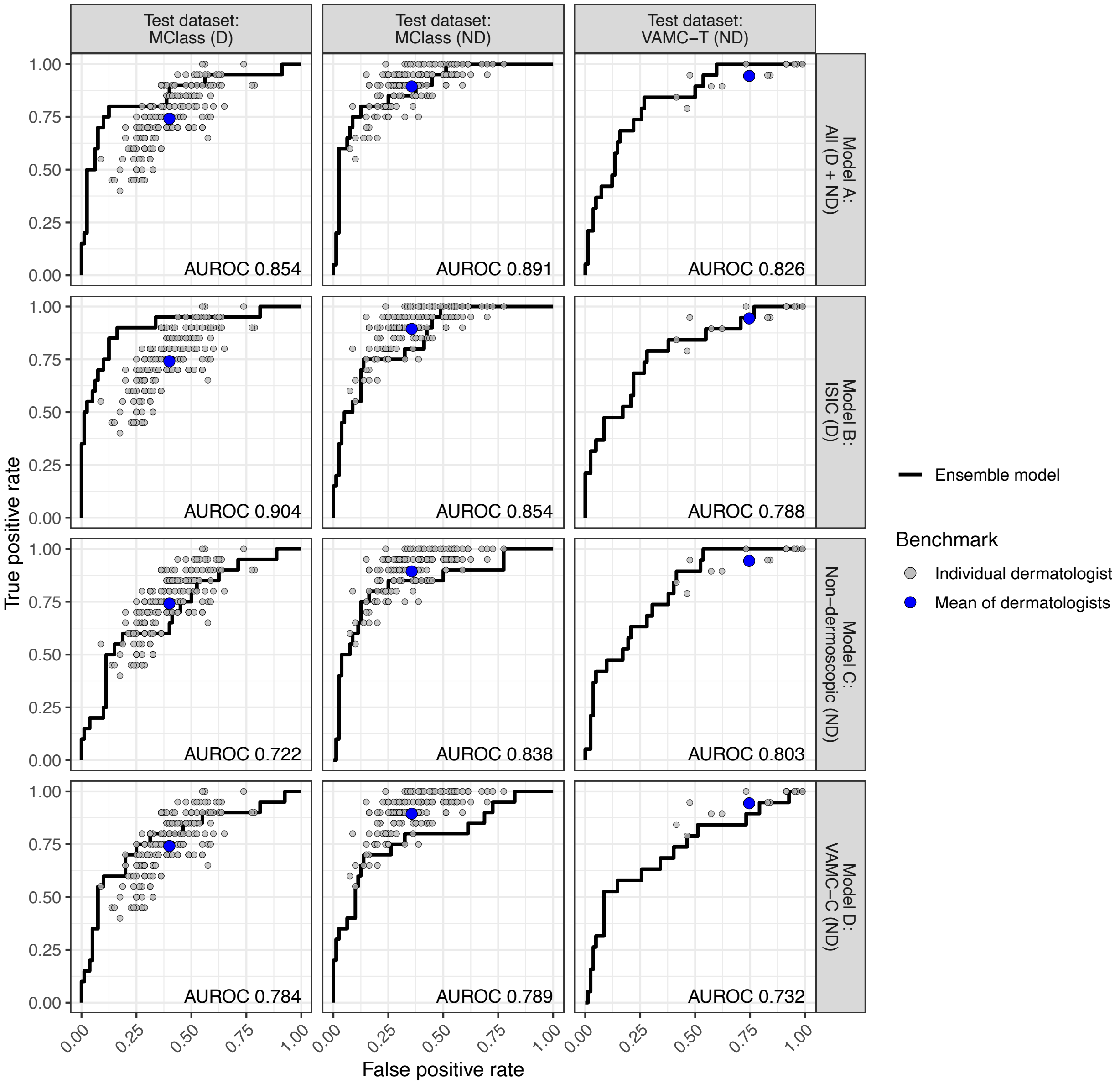

Supplementary Figure 14. Some Individual Dermatologists Exceed CNN Model Discrimination Performance for Diagnostic Decision

ROC curves are shown for each standard CNN ensemble model for VAMC-T. True and false positive rates for diagnostic decision (melanoma vs nevus) are shown for mean dermatologists (blue circles) and individual dermatologists (gray circles). AUROC is shown in text for the ensemble model. Abbreviations: AUROC, area under the receiver operating characteristic curve; CNN, convolutional neural network; D, dermoscopic; ND, non-dermoscopic; VAMC-T, Veterans Affairs Medical Center teledermatology.

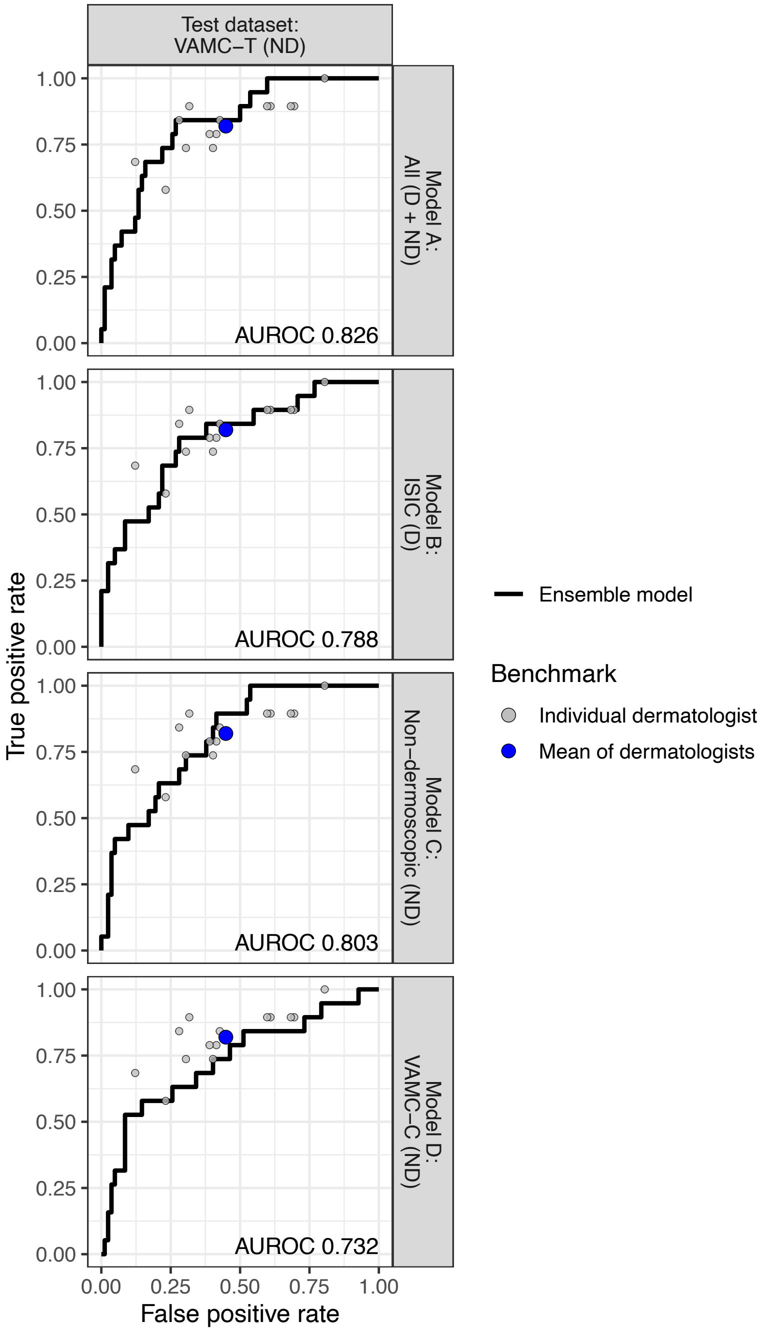

Supplementary References

1. Brinker TJ, Hekler A, Enk AH, et al. A convolutional neural network trained with dermoscopic images performed on par with 145 dermatologists in a clinical melanoma image classification task. *Eur J Cancer*. 2019;111:148-154. doi:10.1016/J.EJCA.2019.02.005
2. Brinker TJ, Hekler A, Enk AH, et al. Deep learning outperformed 136 of 157 dermatologists in a head-to-head dermoscopic melanoma image classification task. *Eur J Cancer*. 2019;113:47-54. doi:10.1016/J.EJCA.2019.04.001
3. He K, Zhang X, Ren S, Sun J. Deep residual learning for image recognition. In: *Proceedings of the IEEE Computer Society Conference on Computer Vision and Pattern Recognition*. Vol 2016-December. IEEE Computer Society; 2016:770-778. doi:10.1109/CVPR.2016.90
4. Hu J, Shen L, Albanie S, Sun G, Wu E. Squeeze-and-Excitation Networks. *Proc IEEE Comput Soc Conf Comput Vis Pattern Recognit*. September 2017:7132-7141. http://arxiv.org/abs/1709.01507. Accessed April 15, 2020.
5. Paszke A, Gross S, Massa F, et al. PyTorch: An Imperative Style, High-Performance Deep Learning Library. In: Wallach H, Larochelle H, Beygelzimer A, d\textquotesingle Alché-Buc F, Fox E, Garnett R, eds. *Advances in Neural Information Processing Systems 32*. Curran Associates, Inc.; 2019:8024-8035. http://papers.neurips.cc/paper/9015-pytorch-an-imperative-style-high-performance-deep-learning-library.pdf.
6. Russakovsky O, Deng J, Su H, et al. ImageNet Large Scale Visual Recognition Challenge. *Int J Comput Vis*. 2015;115(3):211-252. doi:10.1007/s11263-015-0816-y
7. Gutman D, Codella NCF, Celebi E, et al. Skin Lesion Analysis toward Melanoma Detection: A Challenge at the International Symposium on Biomedical Imaging (ISBI) 2016, hosted by the International Skin Imaging Collaboration (ISIC). May 2016. http://arxiv.org/abs/1605.01397. Accessed October 4, 2019.
8. Tschandl P, Rosendahl C, Kittler H. The HAM10000 dataset, a large collection of multi-source dermatoscopic images of common pigmented skin lesions. *Sci Data*. 2018;5(1):180161. doi:10.1038/sdata.2018.161
9. Guo C, Pleiss G, Sun Y, Weinberger KQ. On Calibration of Modern Neural Networks. June 2017. http://arxiv.org/abs/1706.04599. Accessed October 4, 2019.
10. Ziyin L, Wang ZT, Liang PP, Salakhutdinov R, Morency L-P, Ueda M. *Deep Gamblers: Learning to Abstain with Portfolio Theory*.
