# Supplementary material for: Stress testing reveals gaps in clinic readiness of image-based diagnostic artificial intelligence models
Source: NPJ Digit Med. 2021 Jan 21;4:10. doi: 10.1038/s41746-020-00380-6 (PMC7820258; doi:10.1038/s41746-020-00380-6)
Supplement: Supplementary file 2 — Reporting Summary [file 41746_2020_380_MOESM2_ESM.pdf]

nature research

Corresponding author(s): Maria L Wei, MD, PhD
Michael J Keiser, PhD

Last updated by author(s): Nov 26, 2020

Reporting Summary

Nature Research wishes to improve the reproducibility of the work that we publish. This form provides structure for consistency and transparency in reporting. For further information on Nature Research policies, see our Editorial Policies and the Editorial Policy Checklist.

Statistics

For all statistical analyses, confirm that the following items are present in the figure legend, table legend, main text, or Methods section.

n/a	Confirmed	
☐	☒	The exact sample size (*n*) for each experimental group/condition, given as a discrete number and unit of measurement
☐	☒	A statement on whether measurements were taken from distinct samples or whether the same sample was measured repeatedly
☐	☒	The statistical test(s) used AND whether they are one- or two-sided<br>*Only common tests should be described solely by name; describe more complex techniques in the Methods section.*
☒	☐	A description of all covariates tested
☒	☐	A description of any assumptions or corrections, such as tests of normality and adjustment for multiple comparisons
☐	☒	A full description of the statistical parameters including central tendency (e.g. means) or other basic estimates (e.g. regression coefficient) AND variation (e.g. standard deviation) or associated estimates of uncertainty (e.g. confidence intervals)
☐	☒	For null hypothesis testing, the test statistic (e.g. *F*, *t*, *r*) with confidence intervals, effect sizes, degrees of freedom and *P* value noted<br>*Give P values as exact values whenever suitable.*
☒	☐	For Bayesian analysis, information on the choice of priors and Markov chain Monte Carlo settings
☒	☐	For hierarchical and complex designs, identification of the appropriate level for tests and full reporting of outcomes
☒	☐	Estimates of effect sizes (e.g. Cohen's *d*, Pearson's *r*), indicating how they were calculated

Our web collection on statistics for biologists contains articles on many of the points above.

Software and code

Policy information about availability of computer code

Data collection

No software was used for data collection.

Data analysis

R 4.0.0 was used for statistical analysis
For development of convolutional neural networks, Python 3.7.6 was used, with the following packages:
- _libgcc_mutex=0.1=conda_forge
- _openmp_mutex=4.5=1_llvm
- _pytorch_select=0.2=gpu_0
- _r-mutex=1.0.1=anacondar_1
- alabaster=0.7.12=py_0
- art=4.5=py_0
- attrs=19.3.0=py_0
- babel=2.8.0=py_0
- backcall=0.1.0=py_0
- binutils_impl_linux-64=2.34=h53a641e_0
- binutils_linux-64=2.34=hc952b39_18
- black=19.10b0=py37_0
- blas=1.0=mkl
- bleach=3.1.5=pyh9f0ad1d_0
- blosc=1.18.1=he1b5a44_0
- brotlipy=0.7.0=py37h8f50634_1000
- bwidget=1.9.14=0
- bzip2=1.0.8=h516909a_2
- ca-certificates=2020.4.5.1=hecc5488_0

- cairo=1.16.0=hcf35c78_1003
- certifi=2020.4.5.1=py37hc8dfbb8_0
- cffi=1.14.0=py37hd463f26_0
- chardet=3.0.4=py37hc8dfbb8_1006
- coverage=5.1=py37h8f50634_0
- cryptography=2.9.2=py37hb09aad4_0
- cudatoolkit=10.1.243=h6bb024c_0
- cudnn=7.6.5=cuda10.1_0
- curl=7.69.1=h33f0ec9_0
- dbus=1.13.6=he372182_0
- decorator=4.4.2=py_0
- defusedxml=0.6.0=py_0
- dill=0.3.1.1=py37hc8dfbb8_1
- docutils=0.16=py37hc8dfbb8_1
- entrypoints=0.3=py37hc8dfbb8_1001
- expat=2.2.9=he1b5a44_2
- ffmpeg=4.2=h167e202_0
- fontconfig=2.13.1=h86ecdb6_1001
- freetype=2.10.1=he06d7ca_0
- fribidi=1.0.9=h516909a_0
- gcc_impl_linux-64=7.3.0=hd420e75_5
- gcc_linux-64=7.3.0=h553295d_18
- gettext=0.19.8.1=hc5be6a0_1002
- gfortran_impl_linux-64=7.3.0=hdf63c60_5
- gfortran_linux-64=7.3.0=h553295d_18
- giflib=5.2.1=h516909a_2
- glib=2.64.2=h6f030ca_0
- gmp=6.2.0=he1b5a44_2
- gnutls=3.6.5=hd3a4fd2_1002
- graphite2=1.3.13=he1b5a44_1001
- gsl=2.6=h294904e_0
- gst-plugins-base=1.14.5=h0935bb2_2
- gstreamer=1.14.5=h36ae1b5_2
- gxx_impl_linux-64=7.3.0=hdf63c60_5
- gxx_linux-64=7.3.0=h553295d_18
- h5py=2.10.0=nompi_py37h513d04c_102
- harfbuzz=2.4.0=h9f30f68_3
- hdf5=1.10.5=nompi_h3c11f04_1104
- icu=64.2=he1b5a44_1
- imagesize=1.2.0=py_0
- importlib_metadata=1.6.0=0
- ipykernel=5.2.1=py37h43977f1_0
- ipython=7.14.0=py37hc8dfbb8_0
- ipython_genutils=0.2.0=py_1
- jasper=1.900.1=h07fcdf6_1006
- jedi=0.17.0=py37hc8dfbb8_0
- jinja2=2.11.2=pyh9f0ad1d_0
- jpeg=9c=h14c3975_1001
- json5=0.9.0=py_0
- jsonschema=3.2.0=py37hc8dfbb8_1
- jupyter_client=6.1.3=py_0
- jupyter_contrib_core=0.3.3=py_2
- jupyter_core=4.6.3=py37hc8dfbb8_1
- jupyter_nbextensions_configurator=0.4.1=py37_0
- jupyterlab=2.1.2=py_0
- jupyterlab_code_formatter=1.3.1=py_0
- jupyterlab_server=1.1.3=py_0
- krb5=1.17.1=h2fd8d38_0
- lame=3.100=h14c3975_1001
- ld_impl_linux-64=2.34=h53a641e_0
- libblas=3.8.0=14_mkl
- libcblas=3.8.0=14_mkl
- libclang=9.0.1=default_hde54327_0
- libcurl=7.69.1=hf7181ac_0
- libedit=3.1.20170329=hf8c457e_1001
- libffi=3.2.1=he1b5a44_1007
- libgcc-ng=9.2.0=h24d8f2e_2
- libgfortran-ng=7.3.0=hdf63c60_5
- libgomp=9.2.0=h24d8f2e_2
- libiconv=1.15=h516909a_1006
- liblapack=3.8.0=14_mkl
- liblapacke=3.8.0=14_mkl
- libllvm9=9.0.1=he513fc3_1
- libopencv=4.2.0=py37_5
- libpng=1.6.37=hed695b0_1
- libprotobuf=3.11.4=h8b12597_0

- libsodium=1.0.17=h516909a_0
- libssh2=1.8.2=h22169c7_2
- libstdcxx-ng=9.2.0=hdf63c60_2
- libtiff=4.1.0=hc3755c2_3
- libuuid=2.32.1=h14c3975_1000
- libuv=1.34.0=h516909a_0
- libwebp=1.0.2=h56121f0_5
- libxcb=1.13=h14c3975_1002
- libxkbcommon=0.10.0=he1b5a44_0
- libxml2=2.9.10=hee79883_0
- llvm-openmp=10.0.0=hc9558a2_0
- lz4-c=1.9.2=he1b5a44_1
- lzo=2.10=h14c3975_1000
- make=4.3=h516909a_0
- markupsafe=1.1.1=py37h8f50634_1
- mistune=0.8.4=py37h8f50634_1001
- mkl=2019.5=281
- mkl-service=2.3.0=py37h516909a_0
- mock=4.0.2=py37hc8dfbb8_0
- mypy_extensions=0.4.3=py37hc8dfbb8_1
- nbconvert=5.6.1=py37hc8dfbb8_1
- nbformat=5.0.6=py_0
- ncurses=6.1=hf484d3e_1002
- nettle=3.4.1=h1bed415_1002
- ninja=1.10.0=hc9558a2_0
- notebook=6.0.3=py37hc8dfbb8_0
- nspr=4.25=he1b5a44_0
- nss=3.47=he751ad9_0
- numexpr=2.7.1=py37h0da4684_1
- numpy=1.18.4=py37h8960a57_0
- olefile=0.46=py_0
- opencv=4.2.0=py37_5
- openh264=1.8.0=hdbcaa40_1000
- openssl=1.1.1g=h516909a_0
- pandoc=2.9.2.1=0
- pandocfilters=1.4.2=py_1
- pango=1.42.4=h7062337_4
- parso=0.7.0=pyh9f0ad1d_0
- pathspec=0.8.0=pyh9f0ad1d_0
- pcre=8.44=he1b5a44_0
- pcre2=10.34=h2f06484_0
- pexpect=4.8.0=py37hc8dfbb8_1
- pickleshare=0.7.5=py37hc8dfbb8_1001
- pillow=7.1.2=py37h718be6c_0
- pip=20.1=pyh9f0ad1d_0
- pixman=0.38.0=h516909a_1003
- prometheus_client=0.7.1=py_0
- prompt-toolkit=3.0.5=py_0
- pthread-stubs=0.4=h14c3975_1001
- ptyprocess=0.6.0=py_1001
- py-opencv=4.2.0=py37h43977f1_5
- pycm=2.5=py_0
- pycparser=2.20=py_0
- pygments=2.6.1=py_0
- pyopenssl=19.1.0=py_1
- pyrsistent=0.16.0=py37h8f50634_0
- pysocks=1.7.1=py37hc8dfbb8_1
- pytables=3.6.1=py37h9f153d1_1
- python=3.7.6=h8356626_5_cpython
- python-dateutil=2.8.1=py_0
- python_abi=3.7=1_cp37m
- pytorch=1.4.0=cuda101py37h02f0884_0
- pytz=2020.1=pyh9f0ad1d_0
- pyyaml=5.3.1=py37h8f50634_0
- pyzmq=19.0.1=py37hac76be4_0
- qt=5.12.5=hd8c4c69_1
- r-abind=1.4_5=r40h6115d3f_1003
- r-askpass=1.1=r40hcdcec82_2
- r-assertthat=0.2.1=r40h6115d3f_2
- r-backports=1.1.6=r40hcdcec82_2
- r-base=4.0.0=hdca8982_3
- r-base64enc=0.1_3=r40hcdcec82_1004
- r-boot=1.3_25=r40h6115d3f_1
- r-broom=0.5.6=r40h6115d3f_1
- r-callr=3.4.3=r40h6115d3f_1
- r-car=3.0_7=r40h6115d3f_1

- r-cardata=3.0_3=r40h6115d3f_1
- r-caret=6.0_86=r40hcdcec82_2
- r-cellranger=1.1.0=r40h6115d3f_1003
- r-class=7.3_17=r40hcdcec82_1
- r-cli=2.0.2=r40h6115d3f_1
- r-clipr=0.7.0=r40h6115d3f_1
- r-cluster=2.1.0=r40h9bbef5b_3
- r-codetools=0.2_16=r40h6115d3f_1002
- r-colorspace=1.4_1=r40hcdcec82_2
- r-corrplot=0.84=r40_1003
- r-cowplot=1.0.0=r40h6115d3f_2
- r-crayon=1.3.4=r40h6115d3f_1003
- r-curl=4.3=r40hcdcec82_1
- r-data.table=1.12.8=r40hcdcec82_1
- r-dbi=1.1.0=r40h6115d3f_1
- r-dbplyr=1.4.3=r40h6115d3f_1
- r-desc=1.2.0=r40h6115d3f_1003
- r-digest=0.6.25=r40h0357c0b_2
- r-dplyr=0.8.5=r40h0357c0b_1
- r-ellipsis=0.3.0=r40hcdcec82_1
- r-essentials=4.0=r40_2002
- r-evaluate=0.14=r40h6115d3f_2
- r-fansi=0.4.1=r40hcdcec82_1
- r-farver=2.0.3=r40h0357c0b_1
- r-fastmap=1.0.1=r40h0357c0b_1
- r-forcats=0.5.0=r40h6115d3f_1
- r-foreach=1.5.0=r40h6115d3f_1
- r-foreign=0.8_79=r40hcdcec82_0
- r-formatr=1.7=r40h6115d3f_2
- r-fs=1.4.1=r40h0357c0b_1
- r-gdtools=0.2.2=r40h36050f4_1
- r-generics=0.0.2=r40h6115d3f_1003
- r-ggplot2=3.3.0=r40h6115d3f_1
- r-ggpubr=0.3.0=r40h6115d3f_0
- r-ggrepel=0.8.2=r40h0357c0b_1
- r-ggsci=2.9=r40h6115d3f_1003
- r-ggsignif=0.6.0=r40h6115d3f_1
- r-gistr=0.5.0=r40h6115d3f_1
- r-glmnet=3.0_2=r40h9bbef5b_1
- r-glue=1.4.0=r40hcdcec82_1
- r-gower=0.2.1=r40hcdcec82_2
- r-gridextra=2.3=r40h6115d3f_1003
- r-gtable=0.3.0=r40h6115d3f_3
- r-haven=2.2.0=r40hde08347_1
- r-hexbin=1.28.1=r40h9bbef5b_1
- r-highr=0.8=r40h6115d3f_2
- r-hms=0.5.3=r40h6115d3f_1
- r-htmltools=0.4.0=r40h0357c0b_1
- r-htmlwidgets=1.5.1=r40h6115d3f_1
- r-httpuv=1.5.2=r40h0357c0b_2
- r-httr=1.4.1=r40h6115d3f_2
- r-ipred=0.9_9=r40hcdcec82_2
- r-irdisplay=0.7=r40_1002
- r-irkernel=1.1=r40h6115d3f_1
- r-isoband=0.2.1=r40h0357c0b_1
- r-iterators=1.0.12=r40h6115d3f_1
- r-jsonlite=1.6.1=r40hcdcec82_1
- r-kernsmooth=2.23_17=r40hfa343cc_1
- r-knitr=1.28=r40h6115d3f_1
- r-labeling=0.3=r40h6115d3f_1003
- r-later=1.0.0=r40h0357c0b_1
- r-lattice=0.20_41=r40hcdcec82_2
- r-lava=1.6.7=r40h6115d3f_1
- r-lazyeval=0.2.2=r40hcdcec82_2
- r-lifecycle=0.2.0=r40h6115d3f_1
- r-lme4=1.1_23=r40h0357c0b_1
- r-lubridate=1.7.8=r40h0357c0b_1
- r-magrittr=1.5=r40h6115d3f_1003
- r-maps=3.3.0=r40hcdcec82_1004
- r-maptools=0.9_9=r40hcdcec82_1
- r-markdown=1.1=r40hcdcec82_1
- r-mass=7.3_51.6=r40hcdcec82_2
- r-matrix=1.2_18=r40h7fa42b6_3
- r-matrixmodels=0.4_1=r40h6115d3f_1003
- r-mgcv=1.8_31=r40h7fa42b6_1
- r-mime=0.9=r40hcdcec82_1

- r-minqa=1.2.4=r40h6786f55_1005
- r-modelmetrics=1.2.2.2=r40h0357c0b_1
- r-modelr=0.1.7=r40h6115d3f_1
- r-munsell=0.5.0=r40h6115d3f_1003
- r-nlme=3.1_147=r40h9bbef5b_1
- r-nloptr=1.2.2.1=r40h0357c0b_1
- r-nnet=7.3_14=r40hcdcec82_1
- r-numderiv=2016.8_1.1=r40h6115d3f_2
- r-openssl=1.4.1=r40he5c4762_1
- r-openxlsx=4.1.5=r40h0357c0b_0
- r-pbdzmq=0.3_3=r40h559a7a4_1003
- r-pbkrtest=0.4_7=r40h6115d3f_1003
- r-pillar=1.4.4=r40h6115d3f_0
- r-pkgbuild=1.0.8=r40h6115d3f_0
- r-pkgconfig=2.0.3=r40h6115d3f_1
- r-pkgload=1.0.2=r40h0357c0b_1002
- r-plogr=0.2.0=r40h6115d3f_1003
- r-plyr=1.8.6=r40h0357c0b_1
- r-polynom=1.4_0=r40h6115d3f_2
- r-praise=1.0.0=r40h6115d3f_1004
- r-prettyunits=1.1.1=r40h6115d3f_1
- r-proc=1.16.2=r40h0357c0b_1
- r-processx=3.4.2=r40hcdcec82_1
- r-prodlim=2019.11.13=r40h0357c0b_1
- r-progress=1.2.2=r40h6115d3f_2
- r-promises=1.1.0=r40h0357c0b_1
- r-pryr=0.1.4=r40h0357c0b_1004
- r-ps=1.3.3=r40hcdcec82_0
- r-purrr=0.3.4=r40hcdcec82_1
- r-quantmod=0.4.17=r40h6115d3f_1
- r-quantreg=5.55=r40hbf399a0_2
- r-r6=2.4.1=r40h6115d3f_1
- r-randomforest=4.6_14=r40h9bbef5b_1003
- r-rbokeh=0.5.0=r40h6115d3f_1003
- r-rcolorbrewer=1.1_2=r40h6115d3f_1003
- r-rcpp=1.0.4.6=r40h0357c0b_1
- r-rcppeigen=0.3.3.7.0=r40h0357c0b_1
- r-readr=1.3.1=r40h0357c0b_1004
- r-readxl=1.3.1=r40hde08347_4
- r-recipes=0.1.12=r40h6115d3f_1
- r-recommended=4.0=r40_1004
- r-rematch=1.0.1=r40h6115d3f_1003
- r-repr=1.1.0=r40h6115d3f_1
- r-reprex=0.3.0=r40h6115d3f_2
- r-reshape2=1.4.4=r40h0357c0b_1
- r-rio=0.5.16=r40h6115d3f_1002
- r-rlang=0.4.6=r40hcdcec82_0
- r-rmarkdown=2.1=r40h6115d3f_1
- r-rpart=4.1_15=r40hcdcec82_2
- r-rprojroot=1.3_2=r40h6115d3f_1003
- r-rstatix=0.5.0=r40h6115d3f_1
- r-rstudioapi=0.11=r40h6115d3f_1
- r-rvest=0.3.5=r40h6115d3f_1
- r-scales=1.1.0=r40h6115d3f_1
- r-selectr=0.4_2=r40h6115d3f_1
- r-shape=1.4.4=r40_1003
- r-shiny=1.4.0.2=r40h6115d3f_1
- r-showtext=0.8=r40hcdcec82_0
- r-showtextdb=2.0=r40h6115d3f_2
- r-sourcetools=0.1.7=r40he1b5a44_1002
- r-sp=1.4_1=r40hcdcec82_1
- r-sparsem=1.78=r40h9bbef5b_1
- r-spatial=7.3_12=r40hcdcec82_1
- r-squarem=2020.2=r40h6115d3f_1
- r-statmod=1.4.34=r40h6e990d7_1
- r-stringi=1.4.6=r40h0e574ca_2
- r-stringr=1.4.0=r40h6115d3f_2
- r-survival=3.1_12=r40hcdcec82_1
- r-svglite=1.2.3=r40h0357c0b_1
- r-sys=3.3=r40hcdcec82_1
- r-sysfonts=0.8.1=r40hcdcec82_0
- r-systemfonts=0.2.1=r40hc9cbd26_1
- r-testthat=2.3.2=r40h0357c0b_1
- r-tibble=3.0.1=r40hcdcec82_1
- r-tidyr=1.0.3=r40h0357c0b_0
- r-tidyselect=1.0.0=r40h6115d3f_1

```
- r-tidyverse=1.3.0=r40h6115d3f_2
- r-timedate=3043.102=r40h6115d3f_1002
- r-tinytex=0.22=r40h6115d3f_1
- r-ttr=0.23_6=r40hcdcec82_1
- r-utf8=1.1.4=r40hcdcec82_1003
- r-uuid=0.1_4=r40hcdcec82_1
- r-vctrs=0.2.4=r40hcdcec82_1
- r-viridislite=0.3.0=r40h6115d3f_1003
- r-whisker=0.4=r40h6115d3f_1
- r-withr=2.2.0=r40h6115d3f_1
- r-xfun=0.13=r40h6115d3f_1
- r-xml2=1.3.2=r40h0357c0b_1
- r-xtable=1.8_4=r40h6115d3f_3
- r-xts=0.12_0=r40hcdcec82_1
- r-yaml=2.2.1=r40hcdcec82_1
- r-zeallot=0.1.0=r40h6115d3f_1002
- r-zip=2.0.4=r40hcdcec82_1
- r-zoo=1.8_8=r40hcdcec82_0
- readline=8.0=hf8c457e_0
- regex=2020.5.7=py37h8f50634_0
- sed=4.7=h1bed415_1000
- send2trash=1.5.0=py_0
- setuptools=46.2.0=py37hc8dfbb8_0
- simplegeneric=0.8.1=py_1
- snowballstemmer=2.0.0=py_0
- sphinx=3.0.4=py_0
- sphinx_rtd_theme=0.4.3=py_0
- sphinxcontrib-applehelp=1.0.2=py_0
- sphinxcontrib-devhelp=1.0.2=py_0
- sphinxcontrib-htmlhelp=1.0.3=py_0
- sphinxcontrib-jsmath=1.0.1=py_0
- sphinxcontrib-qthelp=1.0.3=py_0
- sphinxcontrib-serializinghtml=1.1.4=py_0
- sqlite=3.30.1=hcee41ef_0
- tensorboardx=2.0=py_0
- terminado=0.8.3=py37hc8dfbb8_1
- testpath=0.4.4=py_0
- tk=8.6.10=hed695b0_0
- tktable=2.10=h555a92e_3
- toml=0.10.0=py_0
- torchvision=0.2.1=py37_1000
- tornado=6.0.4=py37h8f50634_1
- tqdm=4.46.0=pyh9f0ad1d_0
- traitlets=4.3.3=py37hc8dfbb8_1
- typed-ast=1.4.1=py37h516909a_0
- typing_extensions=3.7.4.2=py_0
- tzlocal=2.1=pyh9f0ad1d_0
- wcwidth=0.1.9=pyh9f0ad1d_0
- webencodings=0.5.1=py_1
- wheel=0.34.2=py_1
- x264=1!152.20180806=h14c3975_0
- xorg-kbproto=1.0.7=h14c3975_1002
- xorg-libice=1.0.10=h516909a_0
- xorg-libsm=1.2.3=h84519dc_1000
- xorg-libx11=1.6.9=h516909a_0
- xorg-libxau=1.0.9=h14c3975_0
- xorg-libxdmcp=1.1.3=h516909a_0
- xorg-libxext=1.3.4=h516909a_0
- xorg-libxrender=0.9.10=h516909a_1002
- xorg-renderproto=0.11.1=h14c3975_1002
- xorg-xextproto=7.3.0=h14c3975_1002
- xorg-xproto=7.0.31=h14c3975_1007
- xz=5.2.5=h516909a_0
- yaml=0.2.4=h516909a_0
- yapf=0.29.0=py_0
- zeromq=4.3.2=he1b5a44_2
- zlib=1.2.11=h516909a_1006
- zstd=1.4.4=h6597ccf_3
- pip:
  - alembic==1.0.11
  - appdirs==1.4.3
  - atomicwrites==1.3.0
  - click==7.0
  - cloudpickle==1.2.1
  - configparser==3.7.4
  - coremltools==3.0b1
```

- cox==0.1.post2
- cycler==0.10.0
- databricks-cli==0.8.7
- docker==4.0.2
- flask==1.1.1
- gitdb2==2.0.5
- gitpython==2.1.11
- grpcio==1.28.1
- gunicorn==19.9.0
- idna==2.8
- imageio==2.5.0
- importlib-metadata==0.19
- itsdangerous==1.1.0
- joblib==0.13.2
- jupyter-contrib-nbextensions==0.5.1
- jupyter-highlight-selected-word==0.2.0
- jupyter-latex-envs==1.4.6
- jupyterlab-code-formatter==0.5.0
- jupyterlab-github==1.0.0
- kiwisolver==1.1.0
- lxml==4.4.0
- mako==1.0.13
- matplotlib==3.1.1
- mlflow==1.0.0
- more-itertools==7.2.0
- networkx==2.3
- node==0.9.24
- nodejs==0.1.1
- npm==0.1.1
- odict==1.7.0
- onnx==1.5.0
- onnx-coreml==0.4.0
- optional-django==0.1.0
- packaging==19.1
- pandas==0.23.4
- pluggy==0.12.0
- plumber==1.5
- protobuf==3.9.0
- psutil==5.7.0
- py==1.8.0
- py3nvml==0.2.6
- pyheif==0.4
- pyparsing==2.4.0
- pytest==5.1.0
- python-editor==1.0.4
- python-resize-image==1.1.19
- pywavelets==1.0.3
- querystring-parser==1.2.3
- requests==2.22.0
- rpy2==2.9.4
- scikit-image==0.15.0
- scikit-learn==0.21.2
- scipy==1.3.0
- seaborn==0.9.0
- simplejson==3.16.0
- six==1.10.0
- sklearn==0.0
- smmap2==2.0.5
- sqlalchemy==1.3.5
- sqlparse==0.3.0
- tabulate==0.8.3
- torchsummary==1.5.1
- typing==3.7.4
- typing-extensions==3.7.4
- urllib3==1.25.3
- websocket-client==0.56.0
- werkzeug==0.15.4
- xlrd==1.2.0
- xmltodict==0.12.0
- zipp==0.5.2
- zope-component==4.5
- zope-deferredimport==4.3.1
- zope-deprecation==4.4.0
- zope-event==4.4
- zope-hookable==4.2.0
- zope-interface==4.6.0

- zope-lifecycleevent==4.3
- zope-proxy==4.3.2

For manuscripts utilizing custom algorithms or software that are central to the research but not yet described in published literature, software must be made available to editors and reviewers. We strongly encourage code deposition in a community repository (e.g. GitHub). See the Nature Research guidelines for submitting code & software for further information.

Data

Policy information about availability of data

All manuscripts must include a data availability statement. This statement should provide the following information, where applicable:
- Accession codes, unique identifiers, or web links for publicly available datasets
- A list of figures that have associated raw data
- A description of any restrictions on data availability

The ISIC dataset is available at https://www.isic-archive.com/.
The PH2 dataset is available at https://www.fc.up.pt/addi/ph2%20database.html.
The Melanoma Classification Benchmark is available at https://skinclass.de/mclass/.

The DermNetNZ dataset is available for licensing at https://dermnetnz.org/.
The Dermofit dataset is available for licensing at https://licensing.edinburgh-innovations.ed.ac.uk/i/software/dermofit-image-library.html.

The UCSF and VAMC datasets analyzed during the current study are not publicly available under our Institutional Review Board as they are considered protected health information and cannot be made available to researchers outside this study.

Field-specific reporting

Please select the one below that is the best fit for your research. If you are not sure, read the appropriate sections before making your selection.

☒ Life sciences ☐ Behavioural & social sciences ☐ Ecological, evolutionary & environmental sciences

For a reference copy of the document with all sections, see nature.com/documents/nr-reporting-summary-flat.pdf

Life sciences study design

All studies must disclose on these points even when the disclosure is negative.

Sample size	No power calculations were done. Sample sizes were chosen based on data availability and previous success with developing dermatologist-level convolutional neural networks with similar or smaller sample sizes.
Data exclusions	No data were excluded.
Replication	Forty models were trained in all (4 development datasets x 2 gambler vs. standard training x 5-fold cross-validation), with similar results as described in the manuscript.
Randomization	Randomization is not applicable, given no particular intervention is being studied.
Blinding	Blinding is not applicable, given no particular intervention is being studied.

Reporting for specific materials, systems and methods

We require information from authors about some types of materials, experimental systems and methods used in many studies. Here, indicate whether each material, system or method listed is relevant to your study. If you are not sure if a list item applies to your research, read the appropriate section before selecting a response.

Materials & experimental systems

n/a	Involved in the study	
☒	☐	Antibodies
☒	☐	Eukaryotic cell lines
☒	☐	Palaeontology and archaeology
☒	☐	Animals and other organisms
☐	☒	Human research participants
☒	☐	Clinical data
☒	☐	Dual use research of concern

Methods

n/a	Involved in the study	
☒	☐	ChIP-seq
☒	☐	Flow cytometry
☒	☐	MRI-based neuroimaging

Human research participants

Policy information about studies involving human research participants

Population characteristics	The study population is composed largely of older, white men, given data availability and the prevalence of skin cancer. Details can be found in Supplementary Tables 1 and 2.
Recruitment	For the VAMC and UCSF datasets, participants were recruited over a contiguous period of time from dermatology clinics at the San Francisco Veterans Affairs Medical Center (VAMC) and University of California, San Francisco (UCSF). No exclusion criteria were applied and the potential for selection bias is low. The public "curated" datasets used do not specifically outline their recruitment criteria, and selection bias is a concern, as described in the manuscript.
Ethics oversight	This study was approved by the Institutional Review Board of the University of California, San Francisco.

Note that full information on the approval of the study protocol must also be provided in the manuscript.
